# Structure and Superconductivity of Tin-Containing HfTiZrSn*M* (*M* = Cu, Fe, Nb, Ni) Medium-Entropy and High-Entropy Alloys

**DOI:** 10.3390/ma14143953

**Published:** 2021-07-15

**Authors:** Darja Gačnik, Andreja Jelen, Mitja Krnel, Stanislav Vrtnik, Jože Luzar, Primož Koželj, Marion van Midden, Erik Zupanič, Magdalena Wencka, Anton Meden, Qiang Hu, Sheng Guo, Janez Dolinšek

**Affiliations:** 1 Jožef Stefan Institute, Jamova 39, SI-1000 Ljubljana, Slovenia; darja.gacnik@ijs.si (D.G.); andreja.jelen@ijs.si (A.J.); mitja.krnel@ijs.si (M.K.); stane.vrtnik@ijs.si (S.V.); joze.luzar@ijs.si (J.L.); primoz.kozelj@ijs.si (P.K.); marion.van.midden@ijs.si (M.v.M.); erik.zupanic@ijs.si (E.Z.); magdalena.wencka@ijs.si (M.W.); 2 Faculty of Mathematics and Physics, University of Ljubljana, Jadranska 19, SI-1000 Ljubljana, Slovenia; 3 Institute of Molecular Physics, Polish Academy of Sciences, Smoluchowskiego 17, PL-60-179 Poznań, Poland; 4 Faculty of Chemistry and Chemical Technology, University of Ljubljana, Večna Pot 113, SI-1000 Ljubljana, Slovenia; anton.meden@fkkt.uni-lj.si; 5 Institute of Applied Physics, Jiangxi Academy of Sciences, Changdong Road 7777, Nanchang 330096, China; 6 Industrial and Materials Science, Chalmers University of Technology, SE-41296 Göteborg, Sweden; sheng.guo@chalmers.se

**Keywords:** high-entropy alloys, structure and microstructure, superconductivity

## Abstract

In an attempt to incorporate tin (Sn) into high-entropy alloys composed of refractory metals Hf, Nb, Ti and Zr with the addition of 3*d* transition metals Cu, Fe, and Ni, we synthesized a series of alloys in the system HfTiZrSn*M* (*M* = Cu, Fe, Nb, Ni). The alloys were characterized crystallographically, microstructurally, and compositionally, and their physical properties were determined, with the emphasis on superconductivity. All Sn-containing alloys are multi-phase mixtures of intermetallic compounds (in most cases four). A common feature of the alloys is a microstructure of large crystalline grains of a hexagonal (Hf, Ti, Zr)_5_Sn_3_ partially ordered phase embedded in a matrix that also contains many small inclusions. In the HfTiZrSnCu alloy, some Cu is also incorporated into the grains. Based on the electrical resistivity, specific heat, and magnetization measurements, a superconducting (SC) state was observed in the HfTiZr, HfTiZrSn, HfTiZrSnNi, and HfTiZrSnNb alloys. The HfTiZrSnFe alloy shows a partial SC transition, whereas the HfTiZrSnCu alloy is non-superconducting. All SC alloys are type II superconductors and belong to the Anderson class of “dirty” superconductors.

## 1. Introduction

Within the last two decades, a new concept of metallic alloy design with multiple principal elements in equiatomic or near-equiatomic ratios, termed high-entropy alloys (HEAs), has been launched [1,2,3,4,5]. According to this concept, the high entropy of mixing helps to stabilize random solid solution phases or partially ordered ones with simple crystal structures and small unit cells, in competition with ordered crystalline intermetallic phases and phase-segregated multicomponent mixtures. Tin (Sn) is a post-transition metal in group 14 of the periodic table of elements. It is used in many conventional alloys, most notably tin/lead soft solders, which are typically 60% or more tin, in the manufacture of transparent, electrically conducting films of indium tin oxide in optoelectronic applications and tin-phosphor bronze used for electrical outlets. Another common application for tin is corrosion-resistant tin plating of steel. Because of the low toxicity of inorganic tin, tin-plated steel is widely used for food packaging as tin cans. The addition of tin to the HEAs produced from 3*d*, 4*d,* and 5*d* transition elements has not been widely explored so far. The main problem is the fact that the binary mixing enthalpy of tin with many transition elements is either largely negative, favoring the formation of intermetallic compounds, or positive, promoting phase segregation and dendritic microstructure [6,7,8]. In both cases, random mixing of Sn with other elements on a crystal lattice is compromised and a single-phase random solid solution (a definition of an ideal HEA) is not formed, but a complex multi-phase structure may appear. The disadvantages are also the low melting temperature of tin ($T_{m}=$ 505 K) and its mechanical softness.

Tin resists corrosion from water by forming a protective oxide (passivation) layer at the surfaces of both pewter and other tin alloys [9], which prevents further oxidation. It is thus tempting to employ tin also as one of the components in the HEAs. Most existing studies of the tin-containing HEAs focus on the microstructure and mechanical properties, where it was reported that the Sn addition to the CuMnNiZnSn_x_ [10], CoCrFeNiSn_x_ [11], AlCoCrFeNiSn_x_ [12], CoCuFeNiSn_x_ [13] and CoCrFeMnNiSn_x_ [14] HEAs has resulted in an improved hardness and strength, reduced ductility and improved corrosion resistance. Sn addition has also improved soft magnetic properties of the dual-phase FeCoNi(CuAl)_0.8_Sn_x_ HEAs [15]. New Sn-containing HEAs were searched theoretically in the X-NbTaTiZr system with X = Al, Cr, V, Sn [16], where it was predicted that the Sn-containing alloys are not good candidates to form solid solutions.

In this paper, we explore experimentally the possibility of incorporating tin into HEAs made of refractory metals Hf, Nb, Ti and Zr that are extraordinarily resistant to heat and wear, with the addition of the 3*d* transition metals Cu, Fe and Ni. All alloys were targeted towards equiatomic concentrations of the elements. The aim of this work is (1) to find out whether the selected tin-containing alloys form solid-solution crystalline phases that conform to the definition of a HEA, (2) to characterize compositionally (chemically) and crystallographically the target alloys and (3) to determine their physical properties, with the emphasis on superconductivity. The corrosion-resistance and mechanical properties are not at the focus of this paper and are deferred to another study.

## 2. Materials Synthesis and Characterization

For the preparation of the tin-containing alloys based on the selected refractory metals and the 3*d* transition elements, targeted towards equiatomic concentrations of the elements, we adopted the following strategy. In the first step, a 3-component HfTiZr alloy was synthesized, serving as a reference for all other alloys that were obtained by subsequent addition of other elements to this basic alloy. In the second step, tin was added to obtain a 4-component HfTiZrSn alloy, representing the master tin-containing alloy, to which one element of the Cu, Fe, Nb, and Ni was added in the third step, to obtain four different 5-component tin-containing alloys. These steps refer to the logical sequence of how this series of alloys was designed. In all the alloying processes, all elements were added simultaneously. A short account of the properties of the HfTiZr and HfTiZrSnNb alloys has already been presented in our recent publication [17].

All samples were prepared by arc-melting in an Ar atmosphere. Each ingot was remelted several times and allowed to cool down naturally, so that the materials were in an as-cast state. The starting compositions of the elements were equiatomic. The ingot mass loss after the arc melting was assessed to be less than 0.3%. The chemical composition of most of the alloys was changing slightly from the surface region towards the ingots’ interior. The samples for this study were cut from the central parts of the ingots, where the microstructure was homogeneous.

Properties of the pure elements constituting the investigated alloys (in the order of increasing atomic number) _22_Ti, _26_Fe, _28_Ni, _29_Cu, _40_Zr, _41_Nb, _50_Sn and _72_Hf (the atomic radius r, the type of high-temperature (HT) and room-temperature (RT) structure, the temperature $T_{a}$ of the allotropic transformation to the RT structure, the melting temperature $T_{m}$ and the superconducting transition temperature $T_{C}$) are collected in Table 1 (adapted from [6]). Pure elements Hf, Nb, Ti, and Zr crystallize in a body-centered cubic (bcc) structure just below the solidification temperature. Nb remains bcc down to RT, whereas Hf, Ti, and Zr transform into a hexagonal closely packed (hcp) structure upon cooling. Cu and Ni crystallize in a face-centered cubic (fcc) structure and remain fcc down to RT upon cooling. Iron (Fe) crystallizes first in a bcc structure, undergoes an allotropic transition to fcc at 1665 K and another allotropic transition to the bcc structure at 1184 K, which is then the RT structure. Sn crystallizes in a body-centered tetragonal (bct) structure at the solidification temperature of 505 K (β-tin allotrope), which is metallic and stable at and above RT, whereas the nonmetallic α-tin allotrope is stable below 286 K. According to the atomic size, the elements can be divided into three groups with similar atomic radii, the “small” Cu, Fe and Ni (r= 1.25–1.28 Å), the “medium” Nb, Sn and Ti (r= 1.41–1.46 Å) and the “big” Hf and Zr (r= 1.59 and 1.57 Å, respectively). Regarding the melting temperatures, the one of Sn ($T_{m}=$ 505 K) is much lower than $T_{m}$s of the other elements (which are in the range $T_{m}=$ 1358–2740 K). Superconductivity in the pure metals has been observed for Hf, Nb, Ti, Zr, and Sn ($T_{C}$s are in the range 0.13–9.29 K, with $T_{C}$ of Hf being the lowest and $T_{C}$ of Nb the highest), whereas the Cu, Fe, and Ni metals are non-superconducting [18]. The elements Fe and Ni are magnetic, showing ferromagnetism in the metallic state, whereas other elements are non-magnetic.

The chemical compositions of the investigated alloys within the HfTiZrSn*M* (*M* = Cu, Fe, Nb, Ni) system can be qualitatively predicted by considering the binary (pair) mixing enthalpies $\Delta H_{mix}^{ij}$ of the constituent elements [6,7] (Table 2). The elements Hf, Ti, and Zr mix ideally (their binary mixing enthalpies are all zero), so that the 3-component HfTiZr can be expected to form a random solid solution. Sn has a strong tendency to form intermetallic compounds with Zr ($\Delta H_{mix}^{SnZr}$= –43 kJ mol^−1^), Hf ($\Delta H_{mix}^{SnHf}$= –35 kJ mol^−1^), and Ti ($\Delta H_{mix}^{SnTi}$= –21 kJ mol^−1^), so that the 4-component master alloy HfTiZrSn can be expected to form a multi-phase mixture of intermetallic compounds, with a significant solid solubility (substitution) of the Hf, Ti, and Zr elements. In the 5-component HfTiZrSnFe, the Fe shows strong repulsion (segregation tendency) to Sn ($\Delta H_{mix}^{SnFe}$= 11 kJ mol^−1^), but strong attraction to Hf, Ti, and Zr (the binary mixing enthalpies are between –17 and –25 kJ mol^−1^), so a multi-phase structure can also be expected. Analogously, multi-phase structures are expected for the 5-component HfTiZrSnNi and HfTiZrSnCu. Nb has small binary mixing enthalpies with all other elements in the 5-component HfTiZrSnNb (between –1 and 4 kJ mol^−1^), suggesting its good solid solubility in the alloy and a bit simpler multi-phase structure.

Here, we emphasize that the above compositional and structural predictions are qualitative because they are based on the binary mixing enthalpies only, neglecting the lattice strain energy due to the different atomic sizes that also contribute to the total mixing enthalpy of the alloy. For example, the “ideal” character of the HfTiZr alloy ($\Delta H_{mix}^{ij}=$ 0 for all three pairs of the elements) is compromised by the fact that two “big” elements Hf and Zr are mixed with a “medium-size” element Ti, which produces lattice distortions (lattice strain energy). Minimization of the strain energy promotes the formation of (Hf,Zr)-enriched domains on one side and Ti-enriched domains on the other side.

The structure and chemical composition of the alloys were determined by a combination of X-ray diffraction (XRD), SEM backscattered-electron (BSE) imaging, energy-dispersive X-ray spectroscopy (EDS), and elemental mapping. Due to the complex multi-phase structure of most of the alloys, XRD was used first to determine the number of phases and their crystallographic type by combining search-match, Le Bail fitting and Rietveld refinement of the XRD pattern [19]. Crystallographic data were retrieved from the ICDD PDF–4+ 2020 database. In the next step, the microstructure was determined by SEM BSE imaging, and the chemical composition of the constituent phases was determined via EDS and elemental mapping. In the last step, the XRD analysis was refined according to the BSE and EDS information, to relate the crystallographic structure type of each phase to its actual chemical composition and morphologic appearance in the microstructure. Below, we describe the structural and chemical characterization of each alloy separately. The structural and compositional data, presented in the following, are also collected in Table 3, whereas the EDS elemental maps are presented in the Supplementary Materials.

### 2.1. HfTiZr (HTZ)

**Figure 1:**
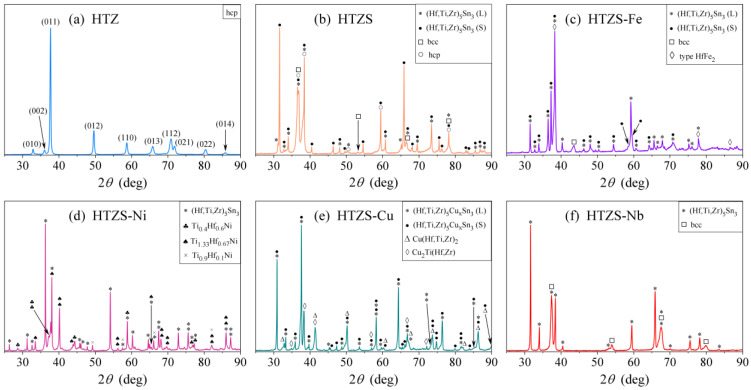
XRD patterns of the six investigated alloys: (**a**) HfTiZr (HTZ), (**b**) HfTiZrSn (HTZS), (**c**) HfTiZrSnFe (HTZS-Fe), (**d**) HfTiZrSnNi (HTZS-Ni), (**e**) HfTiZrSnCu (HTZS-Cu), (**f**) HfTiZrSnNb (HTZS-Nb). For details, see Table 3 and the text. The marks (L) and (S) in some of the legends denote larger and smaller unit cell.

The XRD pattern of the HfTiZr sample (Figure 1a) reveals a single-phase material with a hexagonal close-packed (hcp) structure (space group P6_3_/mmc) and unit cell parameters a= 3.15 Å and c= 4.99 Å. The hcp structure is expected because the Hf, Ti, and Zr pure metals are all hcp at RT. The reflections are quite broad, indicating the small size of the coherently scattering domains, estimated from Scherrer’s equation to be of the order of 50 nm.

**Figure 2:**
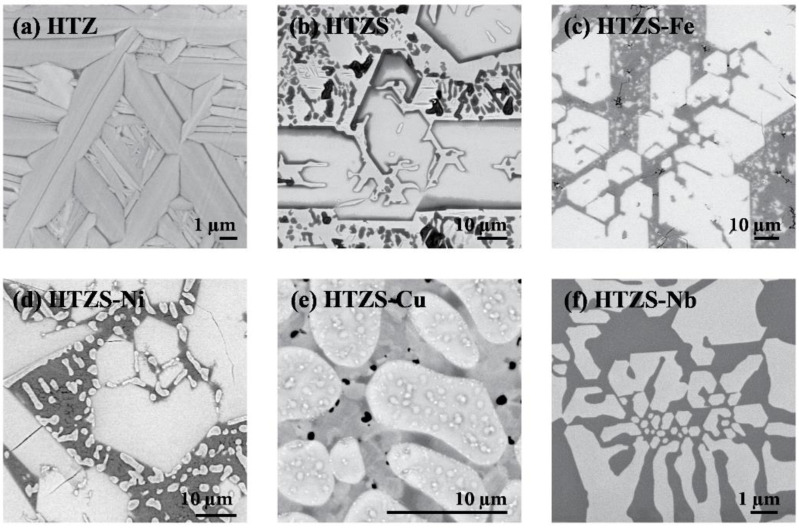
SEM BSE images of (**a**) HfTiZr (HTZ), (**b**) HfTiZrSn (HTZS), (**c**) HfTiZrSnFe (HTZS-Fe), (**d**) HfTiZrSnNi (HTZS-Ni), (**e**) HfTiZrSnCu (HTZS-Cu), (**f**) HfTiZrSnNb (HTZS-Nb). For details, see Table 3 and the text.

The SEM BSE image of a surface prepared by a focused ion beam (FIB) is shown in Figure 2a. A microstructure of µm-size grains with darker edges is evident. EDS-determined composition of the grains (in at.%, rounded to first integers) is Hf_38_Ti_34_Zr_28_, whereas the one of the dark edges is Hf_32_Ti_41_Zr_27_. The experimental grains’ composition is precise to within ±1%, whereas the composition of the dark edges is less precise because of the narrowness of the edges, so some EDS signal from the grains could have also been collected. The dark edges are enriched in the lightest element Ti, whereas the grains are enriched in Hf. This clustering can be understood by the aforementioned tendency of the alloy to minimize the lattice strain energy (a part of the mixing enthalpy) via separating the “medium-size” element Ti from the two “big” elements Hf and Zr. In the following, we shall abbreviate the name of this alloy as HTZ.

### 2.2. HfTiZrSn (HTZS)

The HfTiZrSn master alloy (abbreviated as HTZS) is a multi-phase system. Its XRD pattern is shown in Figure 1b. Some sets of the reflections are sharp and high, whereas others are broad and low. The chemical compositions of the phases and their relation to the microstructure were determined by the EDS analysis of the SEM BSE image shown in Figure 2b. The microstructure consists of large (several 10 µm), faceted grains with straight edges, where the grains’ edges are a bit darker than the interior. The grains are embedded in a light-grey matrix, which also contains a large amount of small inclusions (darker than the matrix), either lamellar or featureless. Some inclusions are very dark. 

The first phase is (Hf,Ti,Zr)_5_Sn_3_, structural type Mn_5_Si_3_ (hexagonal, space group P6_3_/mcm, a= 8.35 Å, c= 5.67 Å), of chemical composition Hf_20_Ti_12_Zr_32_Sn_37_. The Hf, Ti and Zr elements substitute each other randomly at their crystallographic sites, whereas Sn resides at its own crystallographic site. The experimental Sn content of 37 at.% practically equals the theoretical 37.5% for this type of a structure. It is an intermetallic compound, well-crystallized (sharp XRD reflections), with quite large and preferentially oriented crystallites (coherently scattering domains) of several 100 nm dimensions. This phase is found in the large grains, except at their edges.

The second phase corresponds to the grains’ edges and small inclusions in the matrix, except the very dark ones. This phase has the same (Hf,Ti,Zr)_5_Sn_3_ structure as the first phase, but a bit larger unit cell, a= 8.37 Å, c= 5.73 Å and slightly different composition Hf_19_Ti_17_Zr_32_Sn_32_. It contains more Ti at the expense of less Sn, so that the Sn concentration is off-stoichiometric, and the phase composition is less ideal. The XRD reflections of this second phase are broader than those of the first phase, indicating smaller coherently scattering domains (smaller crystallites) due to more disorder.

The third phase is the same hcp (P6_3_/mmc) as the one observed in the HTZ alloy, but with slightly smaller unit cell parameters a= 3.13 Å and c= 4.91 Å, because of a different chemical composition Hf_30_Ti_36_Zr_20_Sn_14_. Broad XRD reflections indicate poorer crystallinity and smaller crystallites of dimensions less than 100 nm. This phase corresponds to the matrix.

The fourth phase is a bcc (Im$\bar{3}$m), a= 3.45 Å. It corresponds to the very dark inclusions in the matrix and is basically a TiZr solid solution with a close to 1:1 stoichiometry, containing also some Hf. Due to the smallness of the inclusions, its chemical composition could not be determined accurately. The XRD reflections’ widths of this phase are intermediate to those of the first and the third phase, indicating also an intermediate size of the crystallites.

### 2.3. HfTiZrSnFe (HTZS-Fe)

The XRD pattern of the HfTiZrSnFe alloy (abbreviated as HTZS-Fe) presented in Figure 1c reveals that the alloy is also composed of four phases. Most XRD reflections are narrow, whereas a small number are considerably broader. The microstructure (Figure 2c) is composed of macroscopically large (10–100 µm), faceted grains with straight edges and homogeneous light-grey color. The grains are embedded in a bit darker grey matrix, which also contains a large amount of small inclusions, most of them lighter grey than the matrix and a few of them considerably darker. A significant amount of cracks is also visible.

The first phase is (Hf,Ti,Zr)_5_Sn_3_, structural type Mn_5_Si_3_ (hexagonal, space group P6_3_/mcm, a= 8.35 Å, c= 5.66 Å), of chemical composition Hf_19_Ti_10_Zr_33_Sn_36_Fe_2_, with the Sn content of 36 at.%, close to the theoretical 37.5% for this type of structure. This phase is found in the entirety of the large grains that are well-crystallized (sharp XRD reflections) and preferentially oriented. It is the same intermetallic phase as that found in the grains of the HTZS master alloy.

The second phase has the same (Hf,Ti,Zr)_5_Sn_3_ structure as the first one, but a bit larger unit cell, a= 8.37 Å, c= 5.69 Å, due to a slightly different (more off-stoichiometric) composition that contains somewhat less Sn and more Ti. The phase composition is less ideal (broader XRD reflections) and more disordered. This phase corresponds to the small light-grey inclusions in the matrix. Due to the smallness of the inclusions, a quantitative determination of the composition by EDS is less precise (and not given here) because some signal from the matrix is also collected.

The third phase corresponds to the matrix. It is a hexagonal Laves phase, type HfFe_2_, P6_3_/mmc, a= 5.10 Å, c= 8.42 Å. Its composition Hf_25_Ti_25_Zr_4_Sn_2_Fe_44_ indicates that Ti, as the intermediate-size atom, is distributed among Hf (large) and Fe (small) sites, so the actual formula is close to (Hf,Ti)(Fe,Ti)_2_ (neglecting the small amounts of Zr and Sn, as their EDS signal could also originate from the surrounding phases). This phase is Fe-rich and contains practically no Sn, indicating that it has formed after all Sn has already been consumed for the formations of the grains of the two (Hf,Ti,Zr)_5_Sn_3_ phases (the first two phases). The XRD peaks of the (Hf,Ti)(Fe,Ti)_2_ phase are broad, indicating poor crystallinity (small crystallites) and a disordered structure.

The fourth phase is a bcc, Im$\bar{3}$m, a= 2.94 Å, containing mostly Ti and up to 10% of other elements, with a statistically occupied single crystallographic site. It corresponds to the dark inclusions in the matrix and due to their smallness, the EDS composition could not be determined precisely.

### 2.4. HfTiZrSnNi (HTZS-Ni)

The XRD pattern of the HfTiZrSnNi alloy (abbreviated as HTZS-Ni) is presented in Figure 1d and reveals a composition of four phases. The microstructure (Figure 2d) shows macroscopically large grains (several 10 to 100 µm) of light grey color, embedded in a darker grey matrix. The matrix also contains a large amount of small bright inclusions and some very dark inclusions.

The first phase is (Hf,Ti,Zr)_5_Sn_3_, structural type Mn_5_Si_3_ (hexagonal, space group P6_3_/mcm, a= 8.40 Å, c= 5.74 Å), of chemical composition Hf_16_Ti_11_Zr_32_Sn_35_Ni_6_, with the Sn content close to the theoretical 37.5% for this type of a structure. This phase is found in the entirety of large grains that are well-crystallized (sharp XRD reflections) and preferentially oriented. The phase occupies the majority of the alloy’s volume and consumes practically all Sn and Zr. It is the same intermetallic phase as also found in the HTZS master alloy and the HTZS-Fe alloy.

The second phase has a cubic structure that is close to Ti_1.33_Hf_0.67_Ni, structural type Nb_2_Ni, space group Fd3m, a= 11.69 Å, of chemical composition Hf_22_Ti_38_Zr_6_Sn_2_Ni_32_. It corresponds to the matrix. The structure is disordered, with Hf and Ti substituting each other statistically at the same crystallographic sites (Nb site as in Nb_2_Ni), whereas Ni resides on its own crystallographic site. The structure also absorbs a small amount of Zr. 

The third phase is orthorhombic, type Ti_0.4_Hf_0.6_Ni, space group Cmcm, a= 3.10 Å, b= 9.67 Å, c= 4.09 Å, of composition Hf_30_Ti_17_Zr_3_Ni_50_. It corresponds to the small bright inclusions in the matrix and possesses the same type of disorder as the second phase.

The fourth phase corresponds to the small dark inclusions in the matrix. It is a monoclinic, close to Ti_0.9_Hf_0.1_Ni, P2_1_/m, a= 2.91 Å, b= 4.02 Å, c= 4.81 Å, β= 98.83°. Due to the smallness of these inclusions, the EDS composition could not be determined.

### 2.5. HfTiZrSnCu (HTZS-Cu)

The XRD pattern of the HfTiZrSnCu alloy (abbreviated as HTZS-Cu) is presented in Figure 1e and reveals that the alloy is also composed of four phases. The XRD reflections are of two types, some of them narrow, while the others are considerably broader. The microstructure (Figure 2e) shows “bean”-shaped grains of dimensions between 10 and 20 µm. These grains are of different shapes than those found in the HTZS master alloy and the HTZS-Fe and HTZS-Ni alloys. The beans also contain many “freckles” of slightly different grey color. The matrix is of a variable, lighter-to-darker grey color, indicating random local compositional changes. There are also some small black inclusions in the matrix.

Both constituents of the grains (the beans and the freckles) belong to the same crystallographic phase, but can readily be resolved by XRD because the reflections of the beans are narrow (larger crystallites, better ordered), while those of the freckles are broader due to smaller crystallites’ size (more disordered structure). The phase is hexagonal, type *M*_5_Cu_x_Sn_3_ (P6_3_/mcm), with the *M* = Hf, Ti, Zr statistically substituting each other at their crystallographic sites, and 0 < x < 1. The compositions of the beans and the freckles are slightly different, which results in slightly different unit cells. The unit cell parameters of the beans are a= 8.47 Å, c= 5.79 Å, while those of the freckles are a= 8.53 Å, c= 5.81 Å (a bit larger unit cell). The chemical composition of the beans and the freckles could not be determined separately because of the too small dimensions of the freckles. The average composition of both is Hf_18_Ti_11_Zr_29_Sn_32_Cu_10_, which can also be written as (Hf,Ti,Zr)_58_Cu_10_Sn_32_, in fair agreement with the *M*_5_Cu_x_Sn_3_ structural type. This structural type is closely related to the Mn_5_Si_3_ (or Hf_5_Sn_3_) type—Cu atoms (partially) occupy the interstices in the Hf_5_Sn_3_ structure.

The matrix is of a slightly variable composition, but of the same structural type in its entirety, as evident from a single set of XRD reflections. The phase is hexagonal, type Cu_2_TiZr, space group P6_3_/mmc, a= 5.15 Å, c= 8.30 Å. In the PDF-4 database, there are several entries indicating that the Cu crystallographic site in such phases can be partially occupied by either Ti or Zr or both and that Ti crystallographic sites can be partially occupied by Cu. In our case, we can expect that Hf and Zr will randomly occupy the Zr sites, while it can be expected, on the basis of the known variations in stoichiometry, that the composition of this phase may vary significantly. This was confirmed by the analysis. The different brighter and darker shades of the matrix originate mainly from the different proportions of the Hf to Zr elements. While in the darker parts the proportion is close to Hf:Zr = 2:1 (its chemical composition is Hf_20_Ti_25_Zr_10_Sn_3_Cu_42_), the brighter parts contain more Hf and less Zr, in addition to more equal concentrations of Cu and Ti (the chemical composition is Hf_25_Ti_33_Zr_7_Sn_2_Cu_33_).

The very dark inclusions in the matrix are Ti-rich and belong to a tetragonal phase, type CuTi_2_ (I4/mmm), a= 3.08 Å, c= 10.90 Å. It can be expected that Hf, Ti and Zr substitute each other statistically. The approximate phase composition determined as Hf_18_Ti_48_Zr_7_Sn_3_Cu_24_ (some EDS signal from the matrix has also been collected) suggests that one or all of these elements may also partially occupy the Cu crystallographic site in the structure. 

### 2.6. HfTiZrSnNb (HTZS-Nb)

The XRD pattern of the HfTiZrSnNb alloy (abbreviated as HTZS-Nb) presented in Figure 1f is less complicated than the XRD patterns of the other investigated 4- and 5-component alloys and can be explained by a two-phase structure. The XRD reflections are of two types, with one set being narrow, while the other is broad. The microstructure (Figure 2f) consists of large grains of lighter-grey color, embedded in a darker-grey matrix.

The first phase is (Hf,Ti,Zr)_5_Sn_3_, structural type Mn_5_Si_3_ (hexagonal, space group P6_3_/mcm, a= 8.35 Å, c= 5.67 Å), of chemical composition Hf_19_Ti_11_Zr_30_Sn_36_Nb_4_, with the Sn content close to the theoretical 37.5% for this type of a structure. This phase corresponds to the grains. It is the same intermetallic phase as found in the grains of the HTZS master alloy and the HTZS-Fe and HTZS-Ni alloys. The XRD reflections are sharp, indicating well-crystallized phase with large crystallites of several 100 nm dimensions.

The second phase corresponds to the matrix, which is bcc, space group Im$\bar{3}$m, a= 3.40 Å. It is a HfTiNb-rich solution of composition Hf_25_Ti_24_Zr_13_Sn_14_Nb_24_, containing also Zr and Sn. The XRD reflections are broad, indicating small crystallites of dimensions well below 100 nm.

## 3. Superconductivity in the HfTiZrSn*M* (*M* = Cu, Fe, Nb, Ni) Alloys

The most spectacular physical property found in the investigated HfTiZrSn*M* (*M* = Cu, Fe, Nb, Ni) alloys is superconductivity, which we have studied experimentally via electrical resistivity, specific heat, magnetic susceptibility, and scanning tunneling spectroscopy. The experimental setup is described in the Methods section.

### 3.1. Electrical Resistivity

**Figure 3:**
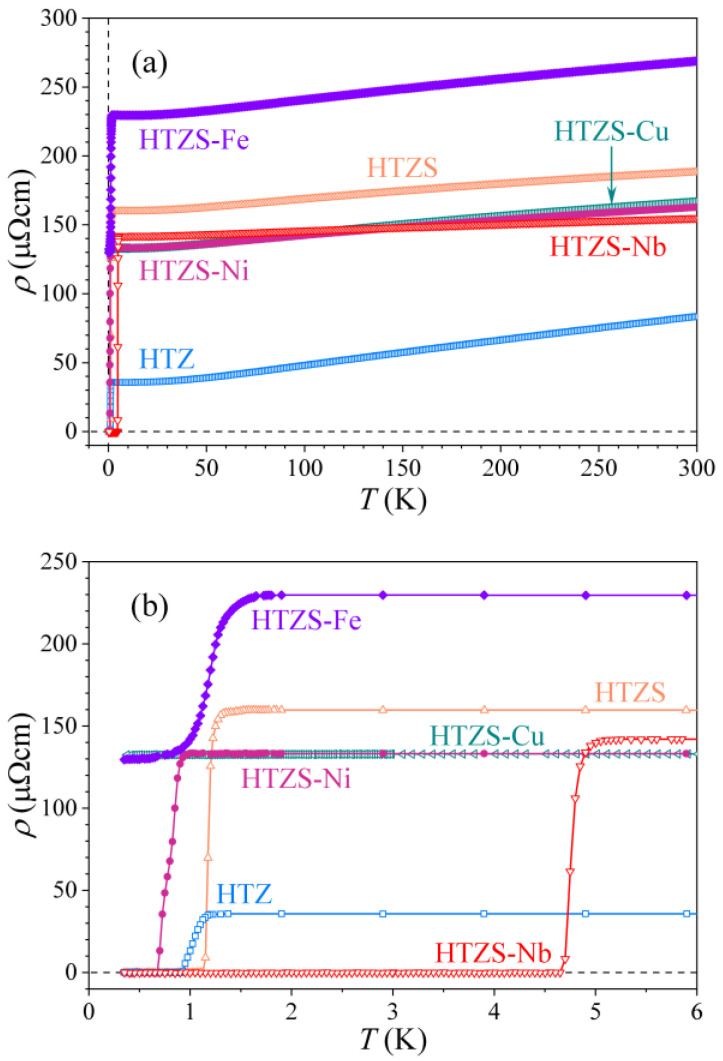
(**a**) Electrical resistivity of the six investigated alloys in the temperature range 300–0.35 K in zero magnetic field. (**b**) The resistivities on an expanded temperature scale below 6 K.

The zero-field electrical resistivity of the six investigated alloys in the temperature range 300–0.35 K is shown in Figure 3a. The samples HTZ, HTZS, HTZS-Ni and HTZS-Nb undergo a transition to a zero-resistance, superconducting (SC) state at a temperature in the range between about 0.5 and 4.6 K. The sample HTZS-Fe undergoes a partial transition to the SC state at about 2 K, where a fraction of the material becomes SC, the rest remaining in the normal state, whereas the sample HTZS-Cu is non-superconducting. The normal-state resistivities of all samples show a weak temperature dependence with a positive temperature coefficient and quite high RT values in the range 83–270 µΩcm, typical of disordered alloys. In Figure 3b, the resistivities are shown on an expanded temperature scale below 6 K, where the details of the SC transition are seen more clearly. The SC transition temperature $T_{C}^{\rho }$ (where the superscript ρ denotes that it was determined from the resistivity) in a zero magnetic field is defined in a standard way as the temperature at which the resistivity within the transition region reaches half of its normal-state value. The alloys possess the following $T_{C}^{\rho }$ values (given in the brackets): HTZ (1.03 K), HTZS (1.18 K), HTZS-Ni (0.67 K), and HTZS-Nb (4.59 K), whereas the partial SC transition in the HTZS-Fe alloy takes place at $T_{C}^{\rho }=$ 1.88 K. The $T_{C}^{\rho }$ values and the RT resistivities $\rho_{300K}$ are also collected in Table 4.

**Figure 4:**
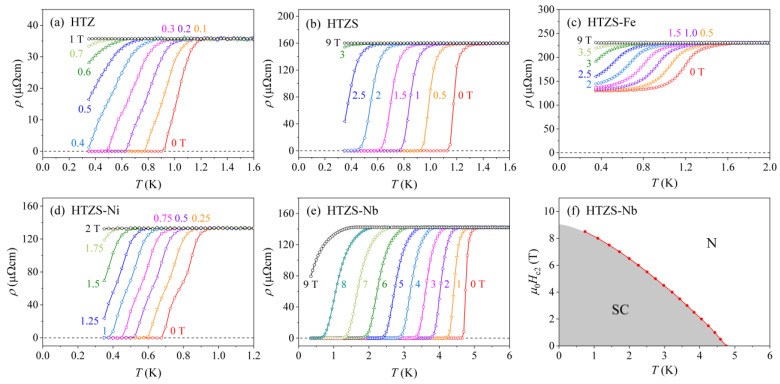
Magnetic-field dependence of the electrical resistivity in the region of the SC transition: (**a**) HfTiZr (HTZ), (**b**) HfTiZrSn (HTZS), (**c**) HfTiZrSnFe (HTZS-Fe), (**d**) HfTiZrSnNi (HTZS-Ni), (**e**) HfTiZrSnNb (HTZS-Nb). (**f**) Temperature dependence of the upper critical field $H_{c2}\left(T\right)$ for the HfTiZrSnNb (HTZS-Nb) alloy, determined from the electrical resistivity (N-normal region, SC-superconducting region).

The magnetic-field dependence of the resistivity in the region of the SC transition is shown in Figure 4. As expected, the SC transition of all samples shifts to lower temperatures with the increasing field. The field-dependent transition temperature $T_{C}^{\rho }\left(H\right)$ of the HTZS-Nb alloy was used to determine the temperature-dependent upper critical field $H_{c2}\left(T\right)$, which for this alloy could not be determined from the specific heat (see later). The $H_{c2}\left(T\right)$ relation is shown in Figure 4f, where the red curve is the fit with the empirical function $H_{c2}\left(T\right)=H_{c2}\left(0\right)\left[1-{\left(T/T_{C}\right)}^{\alpha }\right]$ with α= 1.47 and $\mu_{0}H_{c2}\left(0\right)\approx$ 9.0 T.

### 3.2. Specific Heat

The specific heat C of a superconductor shows a discontinuity (an exothermic peak) at the SC transition due to the formation of Cooper pairs, where the step at $T_{C}$ is $\Delta C\left(T_{C}\right)=C_{s}\left(T_{C}\right)-C_{n}\left(T_{C}\right)$. Here $C_{s}=C_{es}+C_{latt}$ is the specific heat of the SC state, with $C_{es}$ and $C_{latt}$ denoting the electronic and lattice contributions, and $C_{n}=\gamma T+C_{latt}$ is the normal-state specific heat, where γ is the electronic specific heat coefficient [20]. Since $C_{latt}$ does not change at the SC transition, $\Delta C\left(T_{C}\right)=C_{es}\left(T_{C}\right)-\gamma T_{C}$. Chemical and structural (in)homogeneity of the material can be reliably assessed from the height and sharpness of the discontinuity $\Delta C\left(T\to T_{C}\right)={\left(C_{s}-C_{n}\right)}_{T\to T_{C}}$. Homogeneous samples exhibit a high and sharp discontinuity, whereas, in inhomogeneous samples, the discontinuity is broad and smeared over a certain temperature interval. For a perfectly homogeneous material, the slope of the discontinuity on the high-temperature side would be infinitely steep, reflecting the fact that the whole material undergoes the SC transition at precisely the same temperature. A broader discontinuity with a less steep slope on the high-temperature side indicates, on the other hand, a distribution of $T_{C}$s over different parts of the inhomogeneous material.

**Figure 5:**
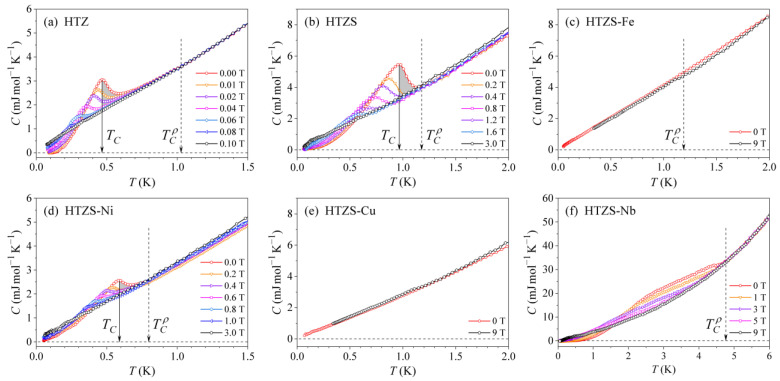
Low-temperature specific heat C(T) curves for selected magnetic fields, measured down to 50 mK: (**a**) HfTiZr (HTZ), (**b**) HfTiZrSn (HTZS), (**c**) HfTiZrSnFe (HTZS-Fe), (**d**) HfTiZrSnNi (HTZS-Ni), (**e**) HfTiZrSnCu (HTZS-Cu), (**f**) HfTiZrSnNb (HTZS-Nb).

The specific heat was measured between RT and 50 mK in magnetic fields between 0 and 9 T. The low-temperature C(T) curves of all samples are presented in Figure 5. The zero-field specific heat of the HTZ, HTZS, and HTZS-Ni samples shows a discontinuity with the peak at 0.47 K, 0.96 K, and 0.59 K, respectively (marked by solid vertical arrows). We associate the temperature of the peak with the SC transition temperature $T_{C}$ and the so-determined $T_{C}$ values are also collected in Table 4. The $T_{C}^{\rho }$ values, determined from the zero-field electrical resistivity, are marked by dashed vertical arrows for comparison. The $T_{C}$ values determined from the specific heat are systematically lower than the $T_{C}^{\rho }$ values. One reason for that is the definition of $T_{C}^{\rho }$ as the temperature where the resistivity reaches half of its normal-state value (so the material is not yet fully superconducting) and the other reason is that zero electrical resistance is measured as soon as there appears a connected path from one electrode to the other through the SC fraction of the inhomogeneous, multi-phase material, even when the SC transition has not yet been completed in all parts of the sample. The $T_{C}$ determined from the peak of the specific heat corresponds, on the other hand, to the temperature where the SC transition has already been completed, so the inequality $T_{C}<T_{C}^{\rho }$ is understandable.

The slope of the discontinuity on the high-temperature side, reflecting the chemical and structural inhomogeneity of the sample, can be conveniently visualized by shading the area between the solid vertical line and the specific heat curve. This has been done in Figure 5 for the HTZ, HTZS and HTZS-Ni samples. The slopes are not steep, indicating a distribution of $T_{C}$s in the inhomogeneous, multi-phase samples.

The zero-field specific heat of the HTZS-Nb sample does not exhibit any pronounced peak-type discontinuity in the specific heat (Figure 5f), though the electrical resistivity clearly demonstrates its SC character below $T_{C}^{\rho }=$ 4.59 K. Instead, the zero-field curve shows an enhancement relative to the normal-state specific heat (represented by the curve in the 9-T field) below 4.6 K. The enhancement decreases in an increasing magnetic field and is no more observed in fields higher than 5 T. This behavior indicates continuously distributed $T_{C}$s below 4.6 K in a strongly inhomogeneous, two-phase material.

The specific heat of the HTZS-Fe and HTZS-Cu samples does not show any anomaly in the low-temperature region (Figure 5c,e). While this is obvious for the non-superconducting HTZS-Cu sample, it is a bit surprising for the HTZS-Fe sample, which undergoes a partial transition to the SC state at $T_{C}^{\rho }=$ 1.88 K (see Figure 3b or Figure 4c). The reason is likely the inhomogeneity of the material, which is so high that the discontinuity is so strongly smeared over a large temperature interval to become unobservable.

**Figure 6:**
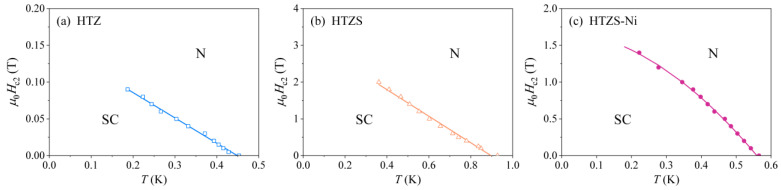
Temperature dependence of the upper critical field $H_{c2}$ determined from the field-dependent specific heat for (**a**) HfTiZr (HTZ), (**b**) HfTiZrSn (HTZS), and (**c**) HfTiZrSnNi (HTZS-Ni) samples (N–normal region, SC–superconducting region). Solid lines in the panels (**a**,**b**) are linear fits, whereas the solid curve in panel (**c**) is the fit with the empirical function $H_{c2}\left(T\right)=H_{c2}\left(0\right)\left[1-{\left(T/T_{C}\right)}^{\alpha }\right]$ with α= 1.88 and $\mu_{0}H_{c2}\left(0\right)=$ 1.8 T.

The investigated SC alloys are all type II superconductors, for which the temperature dependence of the upper critical field $H_{c2}$ can be determined by plotting the temperature of the specific heat maximum as a function of the external magnetic field. In our case this could be done for the HTZ, HTZS, and HTZS-Ni alloys, for which the maxima are well pronounced, whereas it could not be done for the HTZS-Nb alloy due to the not well-developed maximum (for this alloy, the $H_{c2}\left(T\right)$ relation was determined from the field-dependent electrical resistivity and is shown in Figure 4f). The graphs of $\mu_{0}H_{c2}\left(T\right)$ are shown in Figure 6, yielding the extrapolated $\mu_{0}H_{c2}\left(0\right)$ values of the HTZ, HTZS, and HTZS-Ni alloys to be ~0.14, ~3.1, and ~1.8 T. These values are also collected in Table 4.

The γ and $\theta_{D}$ coefficients were determined from the normal-state specific heat, by assuming that the lattice specific heat at temperatures below 10 K can be described by the Debye model, $C_{latt}=\alpha T^{3}$, where $\alpha =12\pi^{4}R/5\theta_{D}^{3}$ is the lattice specific heat coefficient and R is the gas constant. The normal-state specific heat is measured in a magnetic field higher than the upper critical field $H_{c2}$, and the coefficients γ and α are best extracted by presenting the specific heat in a C/T versus $T^{2}$ plot, i.e., $C/T=\gamma +\alpha T^{2}$. The intercept of the vertical axis at T=0 then yields γ, whereas α can be read from the slope of the linear line. For multi-phase alloys, the so-determined γ and α coefficients represent average values over all constituent phases.

The molar fraction x of the SC phase(s) in the alloys can be estimated from the zero-field specific heat [20] (Equation (1)):

(1) $$\frac{C}{T}=x\frac{C_{es}}{T}+\left(1-x\right)\gamma +\alpha T^{2}$$

Upon T→0, the SC electronic specific heat vanishes exponentially, ${\left(C_{es}\right)}_{T\to 0}\propto exp\left(-2\Delta \left(0\right)/k_{B}T\right)\to 0$ (where 2Δ(0) is the energy gap in the electronic density of states (DOS) at the Fermi energy $\varepsilon_{F}$, roughly equal to the energy required to break up a Cooper pair) and so does the ratio ${\left(C_{es}/T\right)}_{T\to 0}\to 0$ [20]. The (C/T) value at T=0 is then used to determine x.

**Figure 7:**
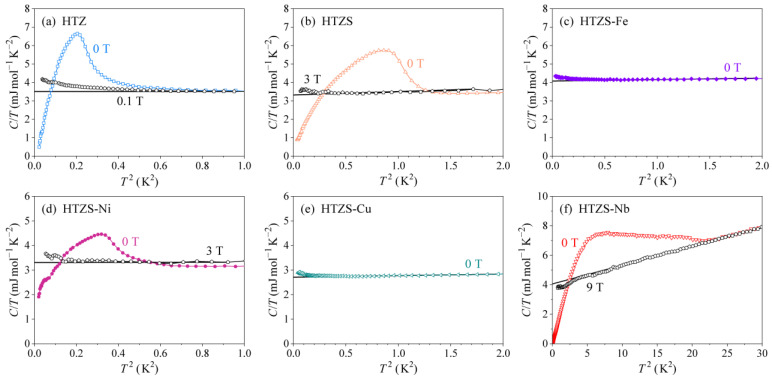
The C/T versus $T^{2}$ plots of the zero-field- and the normal-state specific heat (the latter was measured for the SC samples in a field $H>H_{c2}$) of the samples (**a**) HfTiZr (HTZ), (**b**) HfTiZrSn (HTZS), (**c**) HfTiZrSnFe (HTZS-Fe), (**d**) HfTiZrSnNi (HTZS-Ni), (**e**) HfTiZrSnCu (HTZS-Cu), (**f**) HfTiZrSnNb (HTZS-Nb). Solid lines are $C/T=\gamma +\alpha T^{2}$ fits of the normal-state specific heat.

The C/T versus $T^{2}$ plots of the zero-field- and the normal-state specific heat (the latter was measured in a field $H>H_{c2}$) of the SC samples HTZ, HTZS, HTZS-Ni and HTZS-Nb, as well as the zero-field specific heat of the HTZS-Fe and HTZS-Cu samples are shown in Figure 7. The extracted γ coefficients of all alloys are in the range 2.7–4.1 mJ mol^−1^ K^−2^, whereas the $\theta_{D}$s are in the range 253–303 K (the values for each alloy are given in Table 4). The γ and $\theta_{D}$ values of the alloys are intermediate to the values of the pure constituent metals, which range from $\gamma_{Cu}=$ 0.69 mJ mol^−1^ K^−2^ (the smallest) to $\gamma_{Nb}=$ 7.8 mJ mol^−1^ K^−2^ (the largest) and from $\theta_{D}^{Sn}=$ 199 K (the lowest) to $\theta_{D}^{Ni,Fe}=$ 477 K (the highest) [20]. The determination of the molar fraction x of the SC phase from the T→0 limit of the zero-field specific heat curves is somewhat hampered by the nuclear specific heat $C_{N}$, which becomes observable below about 0.1 K as an upturn of a type $C_{N}=a/T^{2}$, corresponding to the high-temperature side of the nuclear Schottky maximum (that shifts with the magnetic field to higher temperatures) [20]. The presence of $C_{N}$ is clearly seen as an upturn in the T→ 0 limit of the normal-state curves of Figure 7. Since the magnitude of the upturn is similar to the residual ${\left(C/T\right)}_{T\to 0}\to 0$ value of the zero-field curves, we estimate that x≈ 1.

### 3.3. Magnetic Properties

The magnetic state of the investigated alloys was characterized by the magnetization versus the magnetic field curves, M(H), and the temperature-dependent magnetic susceptibility, χ=M/H. Superconductivity is manifested by the Meissner effect, where the magnetic flux density inside the superconductor is zero (B= 0) for the applied field H smaller than the critical field. For the type II superconductors, this condition is met at fields below the lower critical field $H_{c1}$. The volume magnetic susceptibility (which is dimensionless in SI units) of the SC state assumes the ideal diamagnetic value χ= −1 and the isothermal magnetization curve M(H) in the field region $H<H_{c1}$ is consequently linear with a negative slope −1. The M(H) curve exhibits a minimum at $H_{c1}$, followed by an increase towards the normal-state magnetization value that is reached at the upper critical field $H_{c2}$. The M(H) curves taken at different temperatures below $T_{C}$ provide a convenient way to determine the lower critical field $H_{c1}\left(T\right)$, as a function of temperature.

**Figure 8:**
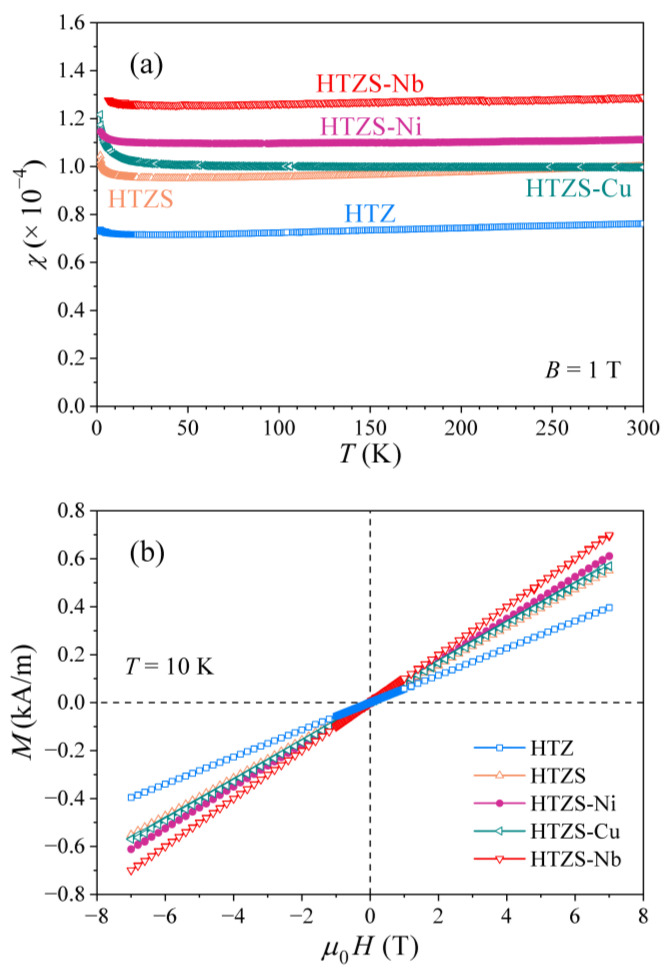
(**a**) Temperature-dependent magnetic susceptibility χ of the HTZ, HTZS, HTZS-Ni, HTZS-Cu, and HTZS-Nb alloys, measured in a field $\mu_{0}H=$ 1 T (the χ(T) curve of the HTZS-Nb is shown only in the normal state down to 5 K). (**b**) The normal-state M(H) curves at T= 10 K.

**Figure 9:**
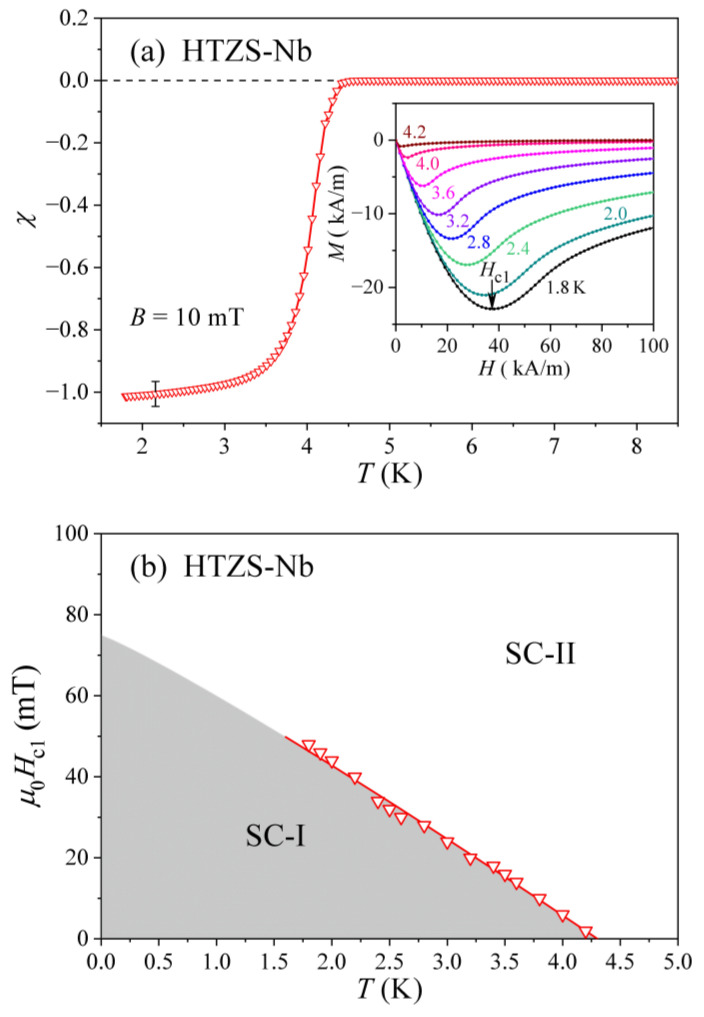
(**a**) Zero-field-cooled magnetic susceptibility χ of the HTZS-Nb alloy in a low magnetic field $\mu_{0}H=$ 10 mT in the region of the superconducting transition. The inset shows isothermal magnetization M(H) at different temperatures, with the arrow denoting the lower critical field $H_{c1}$ at T= 1.8 K. (**b**) The temperature-dependent lower critical field $H_{c1}\left(T\right)$ (SC-I–superconducting region, SC-II–mixed (vortex) state).

The χ(T) and M(H) curves of the investigated alloys were determined between RT and 1.8 K in magnetic fields between 0 and 7 T. The χ(T) curves of the HTZ, HTZS, HTZS-Ni, HTZS-Cu, and HTZS-Nb alloys, measured in a field $\mu_{0}H=$ 1 T are shown in Figure 8a (the χ(T) curve of the HTZS-Nb is shown only in the normal state down to 5 K, whereas the χ(T) at lower temperatures, demonstrating the Meissner effect, is shown on an expanded scale in Figure 9a). The normal-state susceptibilities are all positive and practically temperature-independent (apart from the small Curie upturns observed at low temperatures, originating from extrinsic paramagnetic impurities in the samples). The normal-state M(H) curves presented in Figure 8b are linear in the entire field range, with a positive slope. The positive, temperature-independent susceptibility of a magnitude 10^−4^–10^−5^ and the linear M(H) relation with a positive slope suggest their origin in the Pauli spin paramagnetism of conduction electrons in the electrically conducting nonmagnetic alloys. The nonmagnetic character of the HTZ, HTZS, HTZS-Cu, and HTZS-Nb alloys is straightforward because they are composed of the nonmagnetic elements only, whereas it is not trivial for the HTZS-Ni alloy that contains the magnetic element nickel. However, nickel in this alloy is obviously in a nonmagnetic state, with its magnetic moment screened by the spins of the conduction-electron cloud.

The lowest accessible temperature of 1.8 K in our magnetic experiments has prevented us to observe the Meissner effect for the superconducting HTZ, HTZS, and HTZS-Ni samples because their $T_{C}$s are too low, whereas it could be detected in the HTZS-Nb sample with $T_{C}\approx$ 4.0 K. The Meissner effect in the HTZS-Nb alloy is demonstrated in Figure 9a, where the zero-field-cooled susceptibility χ, measured in a low field $\mu_{0}H=$ 10 mT, is shown in the temperature range below 9 K. χ starts to drop from the paramagnetic value at about 4.3 K and gradually reaches the ideal diamagnetic value of −1 at about 2 K. The gradual drop of the susceptibility over a relatively large temperature interval is another indication of the high structural and chemical inhomogeneity of this alloy, which is in agreement with the specific heat that does not show any pronounced anomaly due to the distribution of $T_{C}$s. The isothermal magnetization M(H) curves for selected temperatures between 1.8 and 4.2 K are shown in the inset of Figure 9a, where the minimum at $H_{c1}$ is clearly observed. The temperature dependence of the lower critical field $H_{c1}\left(T\right)$ is presented in Figure 9b, where a linear extrapolation of the data towards T→ 0 yields $\mu_{0}H_{c1}\left(0\right)\approx$ 70 mT.

**Figure 10:**
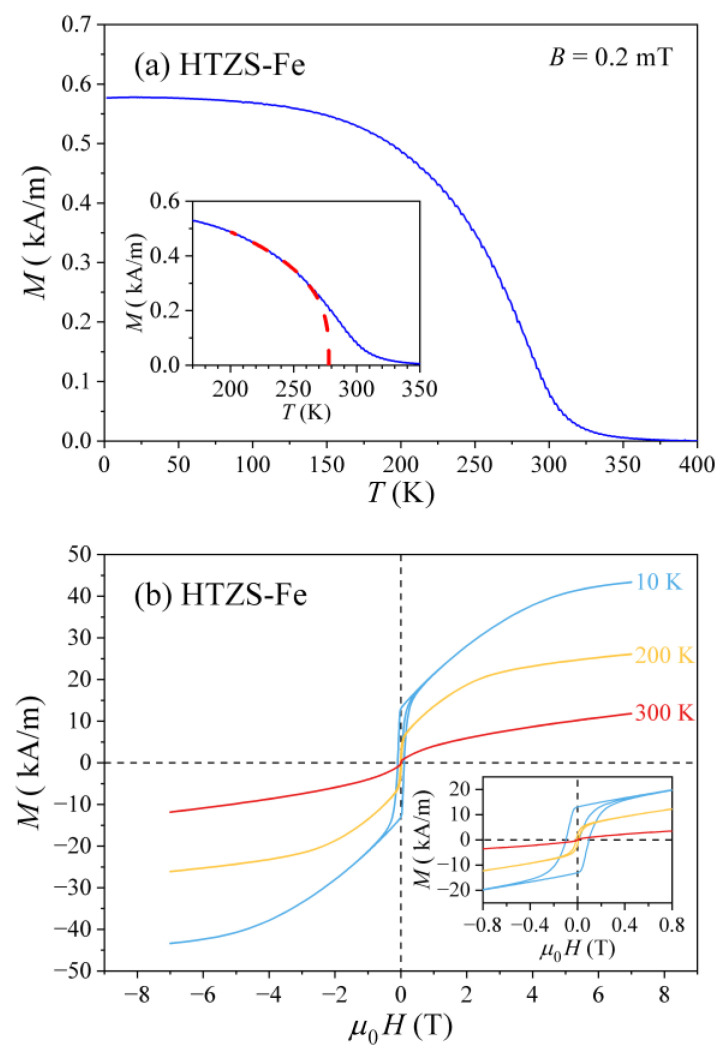
(**a**) Temperature-dependent magnetization M(T) of the HTZS-Fe alloy. The inset shows the fit (dashed curve) within the critical region with the function $M=A{\left(T_{FM}-T\right)}^{\beta }$ (the values of the fit parameters are given in the text). (**b**) The M(H) curves at selected temperatures (indicated in the graph). The ferromagnetic M(H) hysteresis loops are shown on an expanded scale in the inset.

The HTZS-Fe alloy behaves differently from all others. The temperature-dependent magnetization M(T) shown in Figure 10a reveals its ferromagnetic (FM) character below about 300 K, but the FM transition is smeared to higher temperatures due to the inhomogeneity of the material. The FM transition temperature was estimated from the fit in the critical region with the function $M=A{\left(T_{FM}-T\right)}^{\beta }$ (dashed curve in the inset of Figure 10a), which yielded the FM transition temperature $T_{FM}\approx$ 278 K and the critical exponent β= 0.32. The M(H) curves at the selected temperatures of 300, 200, and 10 K are presented in Figure 10b.

The 200 K and 10 K curves are clearly a superposition of an FM part that exhibits hysteresis (shown on an expanded scale in the inset of Figure 10b) and a Curie-like paramagnetic part due to localized paramagnetic centers. The hysteresis curves close up in a field $\mu_{0}H\approx$ 0.3 T that is a typical value for ferromagnets. Both the ferromagnetism and the localized paramagnetism originate from the Fe atoms in the alloy and the two kinds of magnetic behavior are again a consequence of the structural and chemical inhomogeneity of this four-phase alloy.

### 3.4. Superconducting Gap in the HTZS-Nb Alloy Determined by Scanning Tunneling Spectroscopy

Low-temperature scanning tunneling microscopy and spectroscopy (STM and STS) techniques were used to study simultaneously the atomic and electronic structure of the material’s surface with high spatial and energy resolution. By measuring the local density of electronic states (LDOS) on the atomic scale as a function of the STM tip position, one can correlate the electronic properties (e.g., the superconducting band gap) with the surface topography features [21].

The experimental setup and the surface preparation are described in the Methods section. Since the lowest operation temperature of our STM is 1.2 K, only the HTZS-Nb sample with $T_{C}\approx$ 4.0 K could be investigated by STS in the SC state because the $T_{C}$s of other alloys are too low. The HTZS-Nb alloy is a two-phase material, composed of the (Hf,Ti,Zr)_5_Sn_3_ grains embedded in the HfTiNb-rich solid solution matrix phase. Specific heat measurements of the HTZS master alloy, which contains the same kind of the (Hf,Ti,Zr)_5_Sn_3_ grains and becomes superconducting below $T_{C}=$ 0.96 K indicate that only the matrix phase of the HTZS-Nb alloy is superconducting at the STM operating temperature of 1.2 K. Consequently, the superconducting gap determined by STS for this alloy refers to the matrix phase. Since the STM and STS both lack chemical sensitivity, some trial was needed to find the matrix phase at the surface.

**Figure 11:**
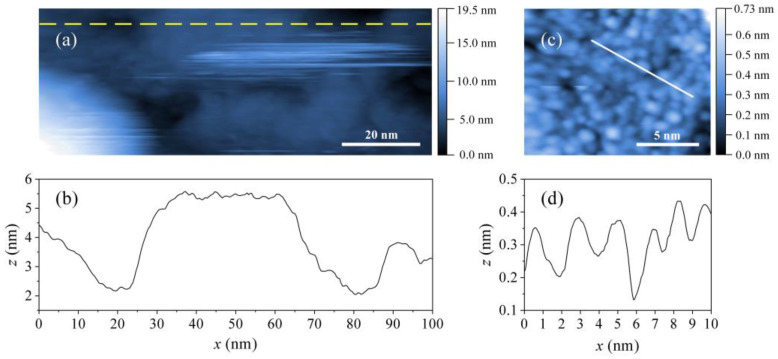
(**a**) The STM topographic image of a 100 × 47 nm^2^ area of the sputtered surface of the HTZS-Nb alloy (the matrix phase) at T= 1.2 K, imaged at 300 mV and 30 pA. (**b**) Height profile over a distance of 100 nm, taken along the dashed yellow line drawn in panel (**a**). (**c**) A 15 × 15 nm^2^ STM image of small clusters on the flat surface (imaged at 100 mV and 60 pA). (**d**) Height profile along the 10-nm white line drawn in panel (**c**).

The STM topographic image of a 100 × 47 nm^2^ sputtered surface at T= 1.2 K is shown in Figure 11a. Some strikes are visible, originating from the remaining mobile impurities on the surface that were displaced by the STM tip during scanning. The height profile taken along the dashed yellow line over the distance of 100 nm is shown in Figure 11b, indicating grain-type structure of the surface with the grains of several tens of nm wide and several nm heights. The tops of the grains are quite flat. The corrugation of the grains on a finer scale was studied along the 10-nm white line in the 15 × 15 nm^2^ image shown in Figure 11c. The height profile (Figure 11d) reveals that the height variations are small, with the maximum variation of about 0.3 nm, meaning that the grain’s surface is practically atomically flat on this scale.

**Figure 12:**
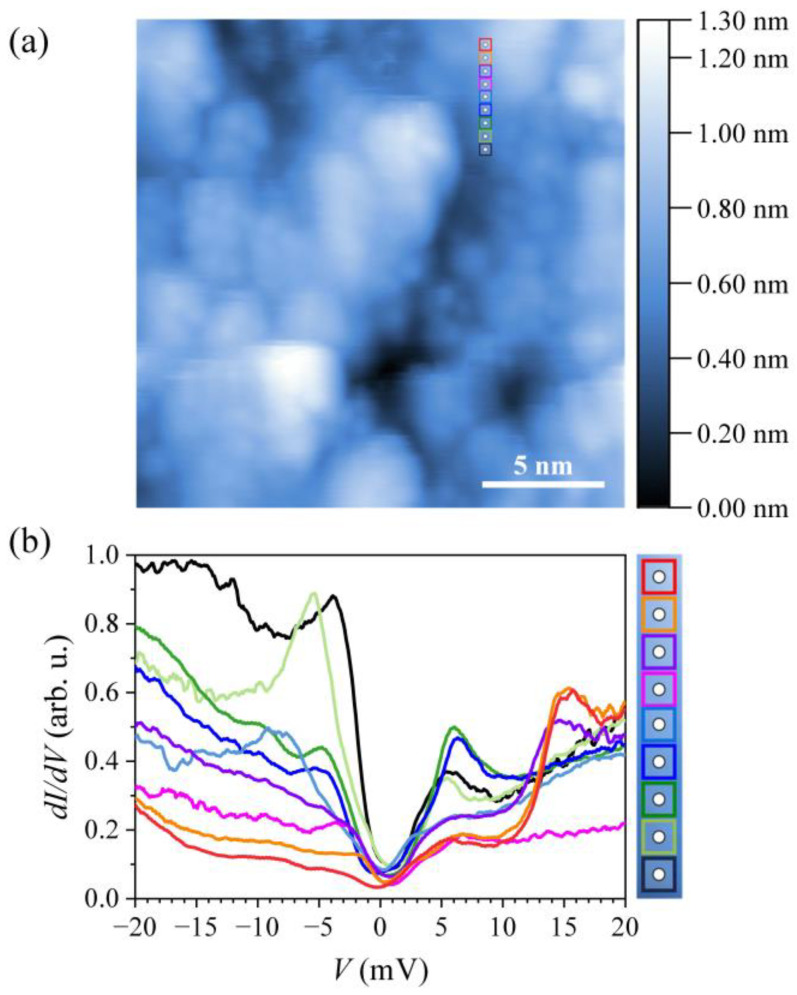
(**a**) A 15 × 15 nm^2^ STM topographic image of the HTZS-Nb sample surface imaged at V= 400 mV and I= 100 pA. The nine dots separated by 0.5 nm (each enclosed by a square of different color) represent locations of the STS measurements. (**b**) dI/dV curves, where the color of each curve matches the color of the square enclosing the point of origin. Local maxima in the dI/dV curves are interpreted as localized states in the LDOS close to the Fermi energy.

To focus on the superconducting gap of expected width about 1–2 meV, we performed STS measurements in a ± 20 meV energy range around the Fermi energy with the modulation voltage amplitude of 0.5 mV for minimum broadening. The dI/dV tunneling conductance curves (where V is the bias voltage between the tip and the sample and I is the tunneling current that is directly proportional to the sample’s LDOS) were measured on a column of nine points separated by 0.5 nm along a line crossing different grains (Figure 12a, where each dot is enclosed by a box of different color). The curves were changing with the position quite noticeably, indicating that the LDOS varies on the atomic scale. This result is plausible because of the atomic-scale chemical (substitutional) disorder in the material. The atomic-scale variation of the dI/dV curves is visible in Figure 12b, where the nine curves are superimposed (the color of each curve matches the color of the square enclosing the point of origin). A common feature of all curves is a superconducting gap of width between 1.0 and 2.5 meV at the Fermi energy. These figures are in agreement with the gap of 2.2 meV reported for the Ta_34_Nb_33_Hf_8_Zr_14_Ti_11_ superconducting HEA [22] and the gap in the pure SC metals like Al, V and Nb, where it is in the range 0.5–1 meV. In some of the dI/dV curves of Figure 12b, localized states in the LDOS close to the Fermi level can be observed as local maxima. When such states are close to $\varepsilon_{F}$, they can “spill” into the superconducting gap due to broadening, making it difficult to precisely determine the size of the gap. For that reason, we estimate the size of the SC gap in the matrix phase of the HTZS-Nb alloy to be about 2Δ≈ 2 meV.

## 4. Discussion

The investigated Sn-containing alloys show common structural and compositional features, by possessing a multi-phase microstructure of large crystalline grains embedded in a matrix that contains also many small inclusions. For the HTZS, HTZS-Fe, HTZS-Ni, and HTZS-Nb alloys, the large grains all possess the (Hf,Ti,Zr)_5_Sn_3_ hexagonal (P6_3_/mcm) partially ordered structure, where Sn resides at one crystallographic site, whereas Hf, Ti and Zr substitute each other statistically at their crystallographic sites. Upon melt solidification, these grains grow preferentially due to the large negative binary mixing enthalpies (attraction) of Sn with the Hf, Ti, and Zr and their growth is terminated once practically all Sn is consumed. The remaining phases are then formed from the “leftover” elements. All constituent phases are crystalline intermetallic compounds, some more and others less disordered. The grains’ structure and composition in the HTZS-Cu alloy are a bit different because some Cu is incorporated into the grains. However, the resulting structure (Hf,Ti,Zr)_5_Cu_x_Sn_3_ (0 < x < 1) remains the same hexagonal P6_3_/mcm as for the (Hf,Ti,Zr)_5_Sn_3_ grains in the other alloys and the a and c lattice parameters of both phases are very similar (see Table 3). The (Hf,Ti,Zr)_5_Cu_x_Sn_3_ grains can thus be considered as a Cu-containing version of the (Hf,Ti,Zr)_5_Sn_3_ grains. In all alloys, these large grains constitute the majority of the samples’ volume. Regarding the actual chemical composition of the (Hf,Ti,Zr)_5_Sn_3_ grains, the EDS analysis presented in Table 3 suggests that in the HTZS-Fe, HTZS-Nb, and HTZS-Ni alloys, the grains contain also a small amount of the transition element Fe, Nb, or Ni, respectively (their compositions are Hf_19_Ti_10_Zr_33_Sn_36_Fe_2_, Hf_19_Ti_11_Zr_30_Sn_36_Nb_4_, and Hf_16_Ti_11_Zr_32_Sn_35_Ni_6_). While a small amount of these elements may indeed be substitutionally incorporated into the grains, it is also likely that their EDS signal is of extrinsic origin, captured in the interaction volume of the electron beam accidentally from the surrounding matrix (e.g., below the grains), so that the grains could, in fact, be pure (Hf,Ti,Zr)_5_Sn_3_. The Cu concentration in the (Hf,Ti,Zr)_5_Cu_x_Sn_3_ grains of composition Hf_18_Ti_11_Zr_29_Sn_32_Cu_10_ is, however, high enough that the Cu incorporation into the grains is undoubted.

The Cu partial incorporation into the (Hf,Ti,Zr)_5_Cu_x_Sn_3_ grains of the HTZS-Cu alloy can be considered to originate from the large negative binary mixing enthalpies (attraction) of Cu with the Zr, Hf, and Ti ($\Delta H_{mix}^{CuZr}$ = −23 kJ mol^−1^, $\Delta H_{mix}^{CuHf}$ = −17 kJ mol^−1^ and $\Delta H_{mix}^{CuTi}$ = −9 kJ mol^−1^). According to the same criterion, and also due to almost the same (small) size of the Ni and Cu atoms, Ni could be expected to incorporate partially into the (Hf,Ti,Zr)_5_Sn_3_ grains of the HTZS-Ni alloy as well, to form (Hf,Ti,Zr)_5_Ni_x_Sn_3_ (the binary mixing enthalpies of Ni with Zr, Hf, and Ti are even more negative, $\Delta H_{mix}^{NiZr}$ = −49 kJ mol^−1^, $\Delta H_{mix}^{NiHf}$ = −42 kJ mol^−1^ and $\Delta H_{mix}^{NiTi}$= −35 kJ mol^−1^). An inspection of the ICDD PDF–4+ 2020 crystallographic database reveals that there indeed exists a Hf_5_NiSn_3_ phase, as the Ni-analogue of the Hf_5_CuSn_3_ phase. The reason why Ni does not incorporate into the (Hf,Ti,Zr)_5_Sn_3_ grains is likely the fact that the Ni stability in the other three phases that constitute the HTZS-Ni alloy is higher so that Ni preferentially binds into those three phases (the orthorhombic Ti_0.4_Hf_0.6_Ni, the cubic Ti_1.33_Hf_0.67_Ni, and the monoclinic Ti_0.9_Hf_0.1_Ni). The Cu, on the other hand, does not have a choice of more stable phases in the HTZS-Cu alloy and consequently picks up the interstitials in the 5:3 phase. This is corroborated also by the ICDD database, which contains much more combinations Hf-Ni than Hf-Cu.

In the structurally and chemically inhomogeneous multi-phase materials, where some of the phases are superconducting and the others not, zero resistance is experimentally measured whenever there exists a connected path from one electrode to the other through the SC fraction of the material. In the investigated alloys, this connected path can be formed through the matrix phase so the structure and chemical composition of the matrix are essential for the observation of superconductivity. Though the large grains constitute the majority of the samples’ volume in all Sn-containing alloys, they are not crucial for the observation of the zero-resistance state. Even if the grains are SC, they do not touch physically each other and hence do not form a connected path through the material. According to the T→ 0 specific heat analysis of the HTZS master alloy, which is SC in the entirety of its volume, the (Hf,Ti,Zr)_5_Sn_3_ grains are superconducting. The Cu-containing grains (Hf,Ti,Zr)_5_Cu_x_Sn_3_ in the HTZS-Cu alloy are, on the other hand, non-superconducting, since this alloy remains in the normal state in the entirety of its volume down to the lowest investigated temperature.

The matrix phases in the HTZ, HTZS, and HTZS-Nb alloys are composed solely of the superconducting elements Hf, Ti, Zr, Sn, and Nb, hence their SC character is not surprising. The matrix in the HTZS-Ni alloy contains, on the other hand, a significant amount of the non-superconducting element Ni (the matrix composition is Hf_22_Ti_38_Zr_6_Sn_2_Ni_32_), but is still SC. This is likely related to the non-magnetic character of Ni in the alloy since the magnetism tends to destroy the Cooper pairs. For the HTZS-Fe alloy, the matrix is the hexagonal Laves phase, type HfFe_2_, which is ferromagnetic below about 300 K. Ferromagnetism inevitably destroys the superconductivity, so the matrix cannot be SC. The partial SC transition in this alloy can be considered to originate from the fact that other constituent phases of this alloy (the large grains and the small inclusions, both of the (Hf,Ti,Zr)_5_Sn_3_ structure, and the dark inclusions that are mostly Ti) accidentally form a partially connected SC path through the material, which reduces the electrical resistivity below $T_{C}\approx$ 1.88 K to about 60% of its normal-state value just above the $T_{C}$, but does not yield zero resistance. In the HTZS-Cu alloy, the presence of a large amount of the non-superconducting element Cu in the compositionally varying matrix (with the Cu concentration fluctuating between 33 and 42 at.%) prevents it to become superconducting.

The structure and morphology of a multi-phase mixture of disordered intermetallic compounds raises the question whether the investigated alloys can be designated as HEAs or not. Following the conventional classification of multi-elemental alloys based on the magnitude of the mixing entropy (that can be written, for equiatomic concentrations of N elements in an ideal solution as $\Delta S_{mix}=RlnN$ [23]), HEAs are defined to possess $\Delta S_{mix}>$ 1.5R, which is achieved by mixing five or more elements (N≥5), whereas the alloys composed of three or four elements belong to the class of medium-entropy alloys (MEAs) with $1R<\Delta S_{mix}<1.5R$ [1,2,3,5,24]. According to these criteria, the 3-component HfTiZr and the 4-component HfTiZrSn alloys would classify as MEAs, whereas the four 5-component HfTiZrSn*M* (*M* = Cu, Fe, Nb, Ni) alloys would be HEAs. However, the alloys are far from random solid solutions and the above classification rules do not apply to the entire alloys, but may be used for the classification of the constituent intermetallic phases. The (Hf,Ti,Zr)_5_Sn_3_ grains are a partially ordered solid solution phase, which satisfies the condition for an MEA. Similar considerations classify the (Hf,Ti,Zr)_5_Cu_x_Sn_3_ grains in the HTZS-Cu alloy as an MEA as well. Regarding the matrices, the disordered solid-solution hcp phases in the 3-component HTZ and the 4-component HTZS (of composition Hf_38_Ti_34_Zr_28_ and Hf_30_Ti_36_Zr_20_Sn_14_, respectively) belong to the class of MEAs, whereas the disordered bcc solid-solution matrix of the 5-component HTZS-Nb of composition Hf_25_Ti_24_Zr_13_Sn_14_Nb_24_ classifies as a HEA. The matrix phases of the HTZS-Fe (the hexagonal Laves phase, type HfFe_2_), the HTZS-Ni (cubic, close to Ti_1.33_Hf_0.67_Ni, type Nb_2_Ni) and the HTZS-Cu alloy (hexagonal, Cu_2_Ti(Hf,Zr) type) are all partially ordered intermetallic compounds that can be classified as MEAs. The investigated Sn-containing alloys can thus be classified as mixtures of MEA phases, whereas the two-phase HTZS-Nb alloy is a mixture of an MEA and a HEA phase. The appropriateness of using the denotations “HEA” and “MEA” for such multi-phase systems can be debated; in particular, whether the denotation “compositionally complex alloys–CCA” is more appropriate, but since it is a common practice in the literature to use the former denotations, we conform to this common trend.

Despite the presence of a huge amount of chemical (substitutional) and topological disorder, the appearance of superconductivity in the HTZ, HTZS, HTZS-Nb, and HTZS-Ni alloys is not surprising. It is known that superconductivity is often insensitive to enormous quantities of chemical and physical imperfections, so disordered crystals and amorphous materials frequently possess SC transition temperatures close to the composition-averaged SC temperature of the constituent elements [25,26]. Electron scattering by quenched chemical or physical defects is elastic, and the electrons in a strongly disordered system are scattered at an extremely rapid rate. Under such conditions, superconductivity is described by the Anderson theory of “dirty” superconductors [27], which states that the theory of Bardeen, Cooper, and Schrieffer (BCS) in the dirty superconductor limit is even more “nearly” correct than it is for pure superconductors.

Recent research of the HEA superconductors [28] suggests that they behave differently from the conventional, metals-based superconductors, copper-oxide (high-$T_{C}$) superconductors, and Fe-based superconductors (iron pnictides), so HEA superconductors may constitute a new class of superconducting materials and represent a special topic in the physics of highly disordered metals and alloys. According to the chemical/structural classification by Sun and Cava [28], the HEA superconductors are divided into four classes. Type-A HEA superconductors consist of the early transition metals with the bcc structure and small unit cells, with the representative examples Ta-Nb-Hf-Zr-Ti [22,29,30,31,32,33] and Nb-Hf-Zr-Ti-V [34]. Type-B HEA superconductors are composed of the 4*d* and 5*d* transition metals (early and late), with the representatives (HfTaWIr)_1−*x*_Re*_x_*, (ZrNb)_1−*x*_(MoReRu)*_x_* and (HfTaWPt)_1−*x*_Re*_x_* and *x* < 0.6. [35]. At high *x* values, these HEAs crystallize on a larger bcc lattice (α-Mn type), while HEAs with lower *x* values crystallize in a mixture of smaller bcc and hcp lattices. Type-C HEA superconductors are composed of the early and late transition metals, crystallizing on a small CsCl-type lattice, with the representative example Sc-Zr-Nb-Ta-Rh-Pd [36]. Type-D HEA superconductors crystallize on an hcp lattice and their representative is Re-Nb-Ti-Zr-Hf [37]. All currently known SC HEAs exhibit type-II superconductivity. The transition temperatures $T_{C}$ are limited to the range below 10 K, and there is a general tendency that the $T_{C}$s of HEAs containing larger fractions of the elements with higher $T_{C}$s (i.e., Nb with $T_{C}=$ 9.2 K, V with $T_{C}=$ 5.3 K and Ta with $T_{C}=$ 4.4 K) are higher, a feature that is characteristic also of the conventional, metals-based SC alloys. $T_{C}$s of the HEA superconductors are intermediate between those of simple binary alloys and amorphous superconductors. For a fixed valence electron concentration, the $T_{C}$s increase with an increasing disorder. HEA superconductors are robust against volume shrinkage under externally applied pressure and the $T_{C}$ saturates to a constant value at a critical pressure [28].

Our investigated Sn-containing SC alloys formally do not belong to any of the above four types of HEA superconductors because they contain a post-transition element Sn. In addition, out of all constituent intermetallic phases, only the matrix of the HTZS-Nb alloy classifies as a HEA, while the other phases are MEAs. Yet the investigated alloys conform to the general trend that their $T_{C}$s increase with the increasing fraction of the elements with higher $T_{C}$ in the matrix phase (in this case Nb with $T_{C}=$ 9.2 K and Sn with $T_{C}=$ 3.7 K). The HTZS-Nb alloy with the matrix composition Hf_25_Ti_24_Zr_13_Sn_14_Nb_24_ consequently shows the highest $T_{C}\approx$ 4.0 K, followed by the HTZS alloy with the matrix composition Hf_30_Ti_36_Zr_20_Sn_14_ and $T_{C}=$ 0.96 K. The HTZ and HTZS-Ni alloys possess zero or a negligible amount of Sn in the matrix and their $T_{C}$s (0.47 and 0.59 K, respectively) are then governed by the $T_{C}$s of Hf, Ti and Zr (see Table 1). For each alloy, the multi-phase microstructure and the high degree of chemical and topological disorder result in a distribution of the $T_{C}$ values over a certain temperature interval, experimentally manifested in the gentle high-temperature slope of the discontinuity in the zero-field specific heat curves. Other details of the SC transition (the exponentially vanishing zero-field specific heat in the T→0 limit, the ~2 meV width of the SC gap in the electronic DOS at the Fermi energy for the HTZS-Nb alloy and the values and temperature-dependence of the lower and upper critical fields) do not appear qualitatively different from other metals-based superconducting alloys involving the 3*d*, 4*d,* and 5*d* transition metals, so the investigated Sn-containing alloys can be considered to be close to a BCS type. The superconductivity in these alloys is extremely robust with respect to all kinds of structural and chemical disorder.

## 5. Conclusions

The investigated Sn-containing alloys are multi-phase mixtures of intermetallic compounds, with some phases more and others less topologically and chemically disordered. A common feature of all alloys is a microstructure of large crystalline grains embedded in a matrix that contains also many small inclusions. For the HTZS, HTZS-Fe, HTZS-Nb, and HTZS-Ni alloys, the large grains possess the (Hf,Ti,Zr)_5_Sn_3_ hexagonal (P6_3_/mcm) partially ordered structure, whereas, in the HTZS-Cu alloy, some Cu is also incorporated into the grains. A superconducting state was observed in the HTZ, HTZS, HTZS-Nb, and HTZS-Ni alloys, the HTZS-Fe alloy shows a partial SC transition, whereas the HTZS-Cu alloy is non-superconducting. The zero resistance of the alloys is determined by the structure and chemical composition of the matrix phase. The strongly chemically and topologically disordered HTZ, HTZS, HTZS-Nb, and HTZS-Ni superconducting alloys are all type II superconductors and belong to the Anderson class of “dirty” superconductors, by exhibiting an extreme robustness of the superconductivity to all kinds of structural and chemical disorder. Due to the inclusion of the post-transition element Sn, they formally do not belong to any of the four different types of the HEA superconductors in the classification by Sun and Cava [28]. Experimental details of the superconductivity are not qualitatively different from other metals-based superconducting alloys involving 3*d*, 4*d*, and 5*d* transition metals, so that the investigated Sn-containing SC alloys can be considered to be close to a BCS type.

## 6. Methods

The XRD diffraction patterns were recorded in reflection mode using a PANalytical X’Pert PRO MPD X-ray powder diffractometer equipped with a primary monochromator delivering pure Cu Kα_1_ radiation (λ= 1.54056 Å) and a 128-channel position-sensitive silicon multi-strip detector. The 2θ range, the integration steps of a continuous scan and the total counting time per step were 20–90° 2θ, 0.033° 2θ, and 200 s, respectively. Le Bail fitting and Rietveld refinements were performed using Topas Academic Version 6 [19].

The SEM BSE imaging and EDS chemical composition determination and elemental mapping were performed by the scanning electron microscopes JEOL JSM-7600F Schottky FE SEM equipped with INCA Oxford 350 EDS SDD, ThermoFisher Quanta 650 ESEM equipped with EDS Oxford Instruments AZtec Live, Ultim Max SDD 40 mm^2^, and Karl *Zeiss Supra* 35 VP SEM with EDS analysis. The samples’ surfaces were prepared by the FIB Helios NanoLab NL650 FEI dual-beam system.

Magnetic measurements were conducted by a Quantum Design MPMS3 SQUID magnetometer equipped with a 7 T magnet, operating down to 1.8 K temperature. Electrical resistivity and specific heat were measured by a Quantum Design Physical Property Measurement System (PPMS 9T), equipped with a 9 T magnet. The temperatures down to 0.35 K were reached by a ^3^He cryostat, whereas a ^3^He/^4^He dilution refrigerator was used to reach temperatures down to 50 mK. Both cryocoolers are compatible with a PPMS 9T apparatus.

The STM and STS measurements were performed at a temperature of 1.2 K using SPECS JT-STM (SPECS Surface Nano Analysis GmbH, Berlin, Germany) with a base pressure of 10^−11^ mbar. STM images were taken in a constant-current mode. In our measurement method, a positive bias voltage corresponds to tunneling from the occupied states of the tip into the empty states of the sample, while an applied negative bias voltage results in tunneling from the occupied states of the sample into the empty states of the tip. STS measurements were performed using the conventional lock-in technique, by modulating the applied dc bias voltage with a sinusoidal voltage of peak-to-peak amplitude between 0.5 and 20 mV (depending on the desired resolution) and modulation frequency of 733 Hz. The sample’s surface was polished manually on the EcoMet 300 polishing machine using 0.05-µm colloidal silica polishing suspension, ultrasonically cleaned, and fixed to a stainless steel plate using two tantalum strips that were spot-welded to the sample plate parallel to the sputtering direction. Before the STM imaging, the surface was sputtered with Ar+ ions for 10 min at 1000 eV in the preparation chamber with 10^−10^ mbar base pressure. The sputtering parameters were optimized in a way that the maximum amount of water molecules and other impurities were removed from the surface while keeping the surface structure undamaged. Due to the complicated sample’s chemical composition, excessive sputtering could induce uneven removal of different atomic species from the surface or implantation of Ar atoms into the surface. To prevent the surface composition alteration, thermal annealing was not performed. While some mobile impurities still remained on the surface (see Figure 11a), the surface prepared in this way was stable enough to perform STM and STS experiments.

## Figures and Tables

**Table 1:** Properties of the pure elements constituting the investigated alloys [6] (the atomic radius r, the type of high-temperature (HT) and room-temperature (RT) structure, the temperature $T_{a}$ of the allotropic transformation to the RT structure, the melting temperature $T_{m}$, and the superconducting transition temperature $T_{C}$).

	Ti	Fe	Ni	Cu	Zr	Nb	Sn	Hf
r (Å)	1.45	1.26	1.25	1.28	1.59	1.46	1.41	1.57
HT structure(s)	bcc	bcc (1665 K < T < 1809 K) fcc (1184 K < T < 1665 K)	same as RT	same as RT	bcc	same as RT	-	bcc
RT structure	hcp	bcc	fcc	fcc	hcp	bcc	bct	hcp
$T_{a}$ (K)	1150	1184	-	-	1136		-	2013
$T_{m}$ (K)	1941	1809	1726	1358	2125	2740	505	2500
$T_{C}$ (K)	0.39	-	-	-	0.55	9.29	3.72	0.13

**Table 2:** Binary mixing enthalpies (in kJ·mol^−1^) for unlike atom pairs constituting the HfTiZrSn*M* (*M* = Cu, Fe, Nb, Ni) alloys [6,7].

**Hf**	0	0	−35	−17	−21	4	−42
0	**Ti**	0	−21	−9	−17	2	−35
0	0	**Zr**	−43	−23	−25	4	−49
−35	−21	−43	**Sn**	7	11	−1	−4
−17	−9	−23	7	**Cu**			
−21	−17	−25	11		**Fe**		
4	2	4	−1			**Nb**	
−42	−35	−49	−4				**Ni**

**Table 3:** List of the investigated alloys, their abbreviated names, chemical composition, and crystallographic parameters. The column “Appearance” describes morphologic appearance of the phase in the SEM-determined microstructure.

Alloy	Abbrev. Name	Appearance	Composition	Structure
HfTiZr	HTZ	grains interior	Hf_38_Ti_34_Zr_28_	hexagonal close-packed (hcp), P6_3_/mmc, a= 3.15 Å, c= 4.99 Å
		grains’ dark edges	Hf_32_Ti_41_Zr_27_	
HfTiZrSn	HTZS	large grains interior	Hf_20_Ti_12_Zr_32_Sn_37_	hexagonal, (Hf,Ti,Zr)_5_Sn_3_ (Mn_5_Si_3_ type), P6_3_/mcm, a= 8.35 Å, c= 5.67 Å
		large grains edges, small bright inclusions	Hf_19_Ti_17_Zr_32_Sn_32_	hexagonal, (Hf,Ti,Zr)_5_Sn_3_ (Mn_5_Si_3_ type), P6_3_/mcm, a= 8.37 Å, c= 5.73 Å
		matrix	Hf_30_Ti_36_Zr_20_Sn_14_	hcp, P6_3_/mmc, a= 3.13 Å, c= 4.91 Å
		dark inclusions		bcc, TiZr-rich solid solution, Im$\bar{3}$m, a= 3.45 Å
HfTiZrSnFe	HTZS-Fe	large grains	Hf_19_Ti_10_Zr_33_Sn_36_Fe_2_	hexagonal, (Hf,Ti,Zr)_5_Sn_3_ (Mn_5_Si_3_ type), P6_3_/mcm, a= 8.35 Å, c= 5.66 Å
		small bright inclusions	similar to large grains, less Sn, more Ti	hexagonal, (Hf,Ti,Zr)_5_Sn_3_ (Mn_5_Si_3_ type), P6_3_/mcm, a= 8.37 Å, c= 5.69 Å
		matrix	Hf_25_Ti_25_Zr_4_Sn_2_Fe_44_	hexagonal Laves phase, close to (Hf,Ti)(Fe,Ti)_2_, type HfFe_2_, P6_3_/mmc, a= 5.10 Å, c= 8.42 Å
		dark inclusions		bcc, mostly Ti (up to 10% other elements), Im$\bar{3}$m, a= 2.94 Å
HfTiZrSnNi	HTZS-Ni	large grains	Hf_16_Ti_11_Zr_32_Sn_35_Ni_6_	hexagonal, (Hf,Ti,Zr)_5_Sn_3_ (Mn_5_Si_3_ type), P6_3_/mcm, a= 8.40 Å, c= 5.74 Å
		small bright inclusions	Hf_30_Ti_17_Zr_3_Ni_50_	orthorhombic, Ti_0.4_Hf_0.6_Ni, Cmcm, a= 3.10 Å, b= 9.67 Å, c= 4.09 Å
		matrix	Hf_22_Ti_38_Zr_6_Sn_2_Ni_32_	cubic, close to Ti_1.33_Hf_0.67_Ni (Nb_2_Ni type), Fd3m, a= 11.69 Å
		dark inclusions		monoclinic, close to Ti_0.9_Hf_0.1_Ni, P2_1_/m, a= 2.91 Å, b= 4.02 Å, c= 4.81 Å, β= 98.83^o^
HfTiZrSnCu	HTZS-Cu	grains (beans)	Hf_18_Ti_11_Zr_29_Sn_32_Cu_10_	hexagonal, type (Hf,Ti,Zr)_5_Cu_x_Sn_3_, 0 < x < 1, P6_3_/mcm, a= 8.47 Å, c= 5.79 Å
		freckles on beans		hexagonal, type (Hf,Ti,Zr)_5_Cu_x_Sn_3_, 0 < x < 1, P6_3_/mcm, a= 8.53 Å, c= 5.81 Å
		matrix-dark	Hf_20_Ti_25_Zr_10_Sn_3_Cu_42_	hexagonal, type Cu_2_TiZr, P6_3_/mmc, a= 5.15 Å, c= 8.30 Å
		matrix-bright	Hf_25_Ti_33_Zr_7_Sn_2_Cu_33_	
		dark inclusions	~Hf_18_Ti_48_Zr_7_Sn_3_Cu_24_	tetragonal, type CuTi_2_, I4/mmm, a= 3.08 Å, c= 10.90 Å
HfTiZrSnNb	HTZS-Nb	grains	Hf_19_Ti_11_Zr_30_Sn_36_Nb_4_	hexagonal, (Hf,Ti,Zr)_5_Sn_3_ (Mn_5_Si_3_ type), P6_3_/mcm, a= 8.35 Å, c= 5.67 Å
		matrix	Hf_25_Ti_24_Zr_13_Sn_14_Nb_24_	bcc, HfTiNb-rich solid solution, Im$\bar{3}$m, a= 3.40 Å

**Table 4:** Parameters determined from the electrical resistivity (the SC transition temperature $T_{C}^{\rho }$ and the $\rho_{300K\;}$ resistivity) and the specific heat (the SC transition temperature $T_{C}$, the upper critical field $\mu_{0}H_{c2}\left(0\right)$, the electronic specific heat coefficient γ and the Debye temperature $\theta_{D}$) for the investigated alloys.

Alloy	Electrical Resistivity		Specific Heat			
	$T_{C}^{\rho }$ (K)	$\rho_{300K}$ (µΩcm)	$T_{C}$ (K)	$\mu_{0}H_{c2}\left(0\right)$ (T)	γ (mJ mol^−1^ K^−2^)	$\theta_{D}$ (K)
HTZ	1.03	83.5	0.47	~0.14	3.51	303
HTZS	1.18	188.2	0.96	~3.1	3.32	269
HTZS-Fe	1.88 (partial)	268.9	-	-	4.08	289
HTZS-Ni	0.67	162.8	0.59	~1.8	3.31	289
HTZS-Cu	-	166.9	-	-	2.71	278
HTZS-Nb	4.59	155.1	~4.0	~9.0	4.06	253

## Data Availability

The data presented in this study are available on request from the corresponding authors.

## Supplementary Materials

The following are available online at https://www.mdpi.com/article/10.3390/ma14143953/s1. EDS elemental maps of the HfTiZrSn*M* (*M* = Cu, Fe, Nb, Ni) alloys.
