# Supplementary material for: Structure and Superconductivity of Tin-Containing HfTiZrSnM (M = Cu, Fe, Nb, Ni) Medium-Entropy and High-Entropy Alloys
Source: Materials (Basel). 2021 Jul 15;14(14):3953. doi: 10.3390/ma14143953 (PMC8305006; doi:10.3390/ma14143953)

Supplementary Material

Structure and superconductivity of tin-containing HfTiZrSn*M* (*M* = Cu, Fe, Nb, Ni) medium-entropy and high-entropy alloys

Darja Gačnik [1], Andreja Jelen [1], Mitja Krnel [1], Stanislav Vrtnik [1], Jože Luzar [1], Primož Koželj [1,2], Marion van Midden [1], Erik Zupanič [1], Magdalena Wencka [1,3], Anton Meden [4], Qiang Hu [5,*], Sheng Guo [6] and Janez Dolinšek [1,2,*]

[1] Jožef Stefan Institute, Jamova 39, SI-1000 Ljubljana, Slovenia

[2] University of Ljubljana, Faculty of Mathematics and Physics, Jadranska 19, SI-1000 Ljubljana, Slovenia

[3] Institute of Molecular Physics, Polish Academy of Sciences, Smoluchowskiego 17, PL-60-179 Poznan, Poland

[4] University of Ljubljana, Faculty of Chemistry and Chemical Technology, Večna pot 113, SI-1000 Ljubljana, Slovenia

[5] Institute of Applied Physics, Jiangxi Academy of Sciences, Changdong Road 7777, Nanchang 330096, PR China

[6] Industrial and Materials Science, Chalmers University of Technology, SE-41296 Göteborg, Sweden

* Correspondence: q-fei618@qq.com (Q.H.); janez.dolinsek@ijs.si (J.D)

EDS elemental maps of the investigated Sn-containing alloys:

a) HfTiZr (HTZ)

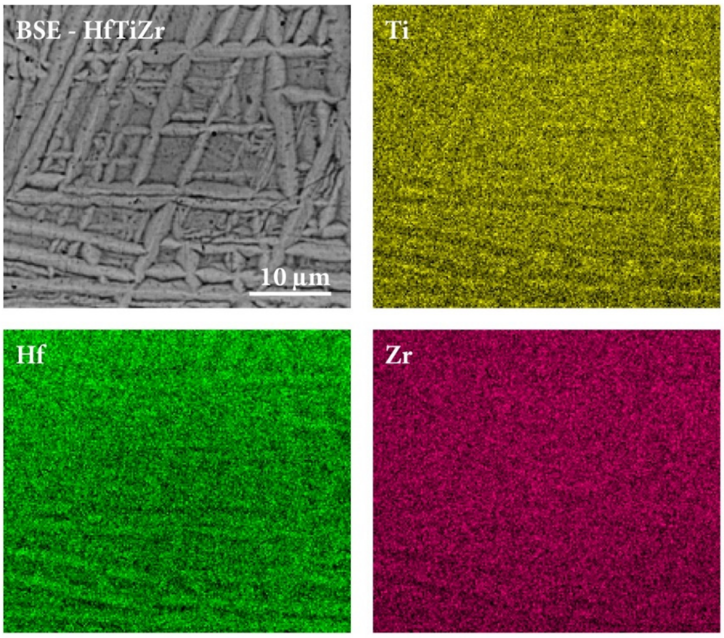

b) HfTiZrSn (HTZS)

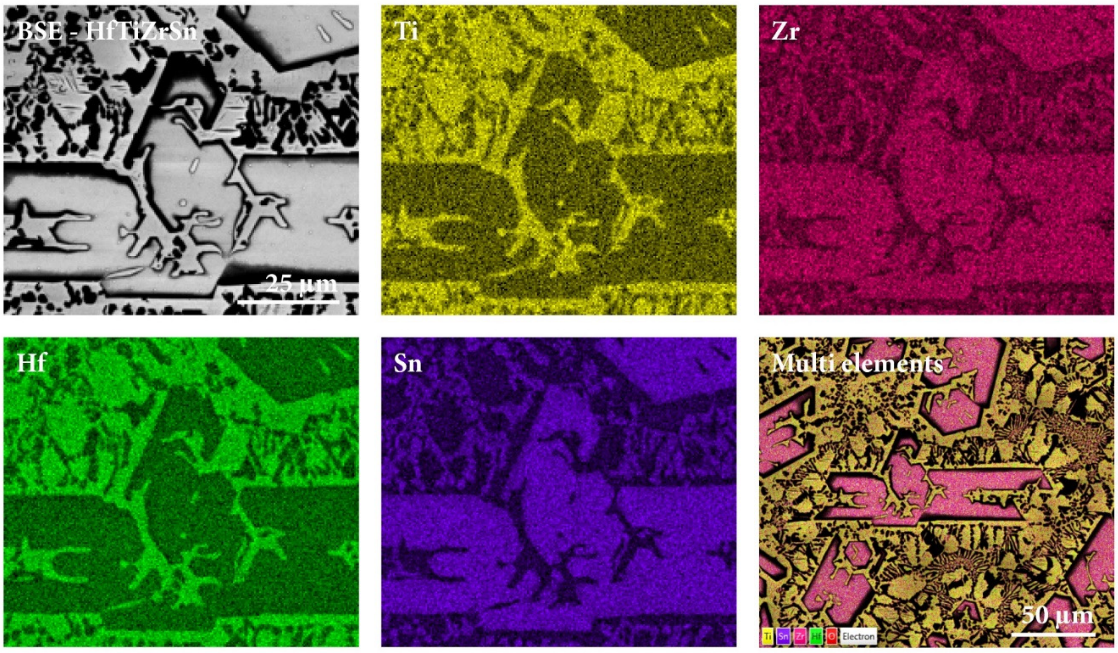

c) HfTiZrSnFe (HTZS-Fe)

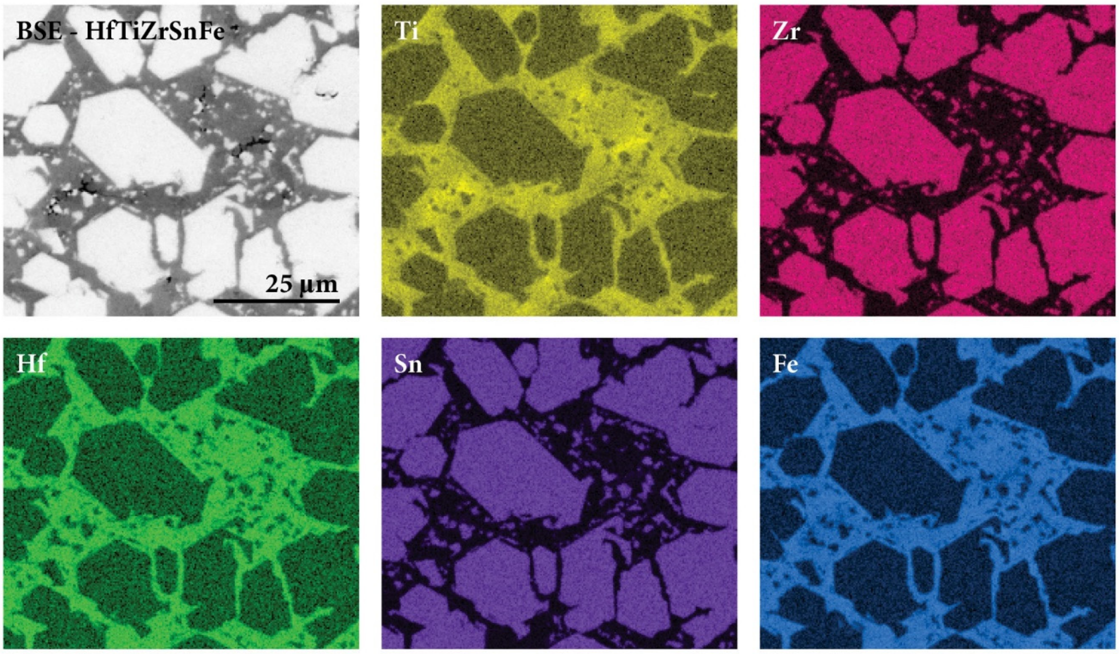

d) HfTiZrSnNi (HTZS-Ni)

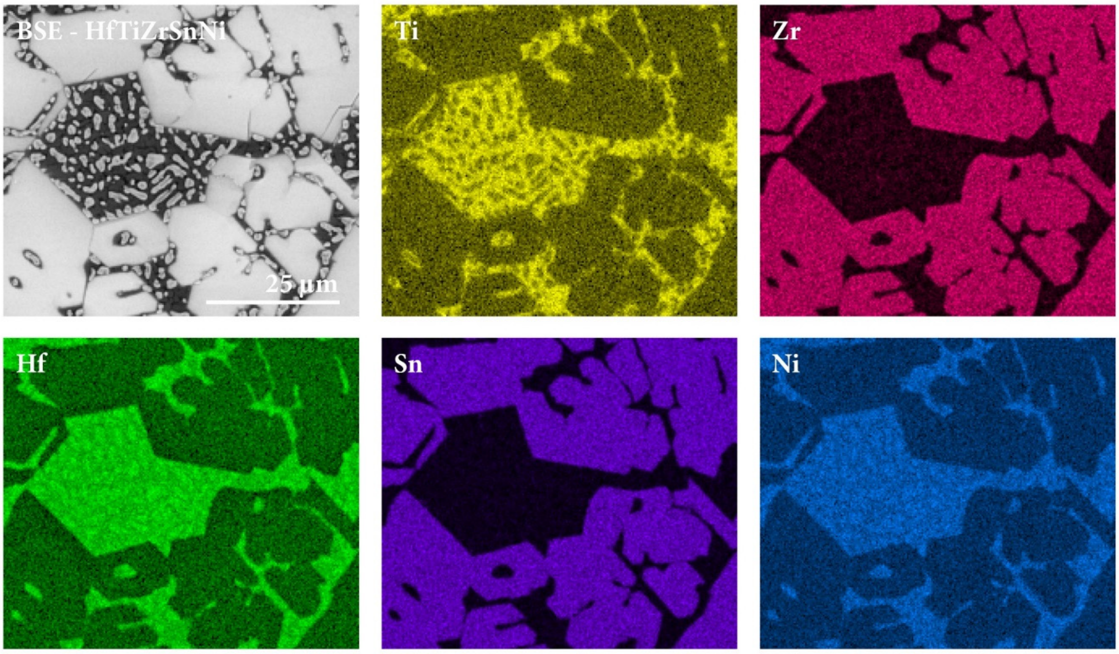

e) HfTiZrSnCu (HTZS-Cu)

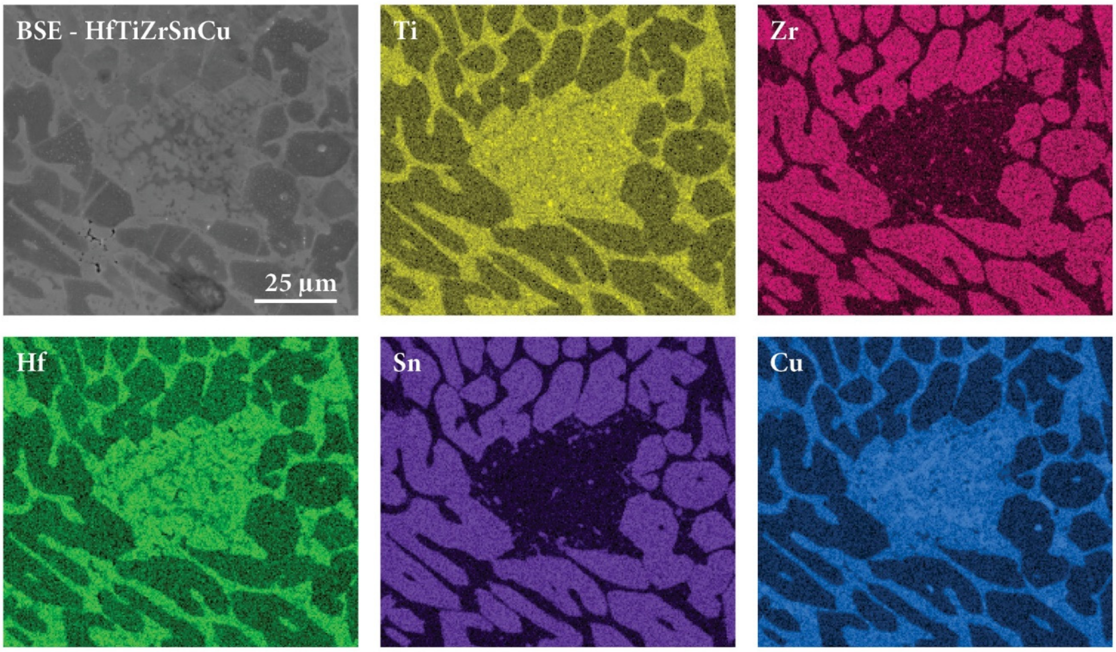

f) HfTiZrSnNb (HTZS-Nb)

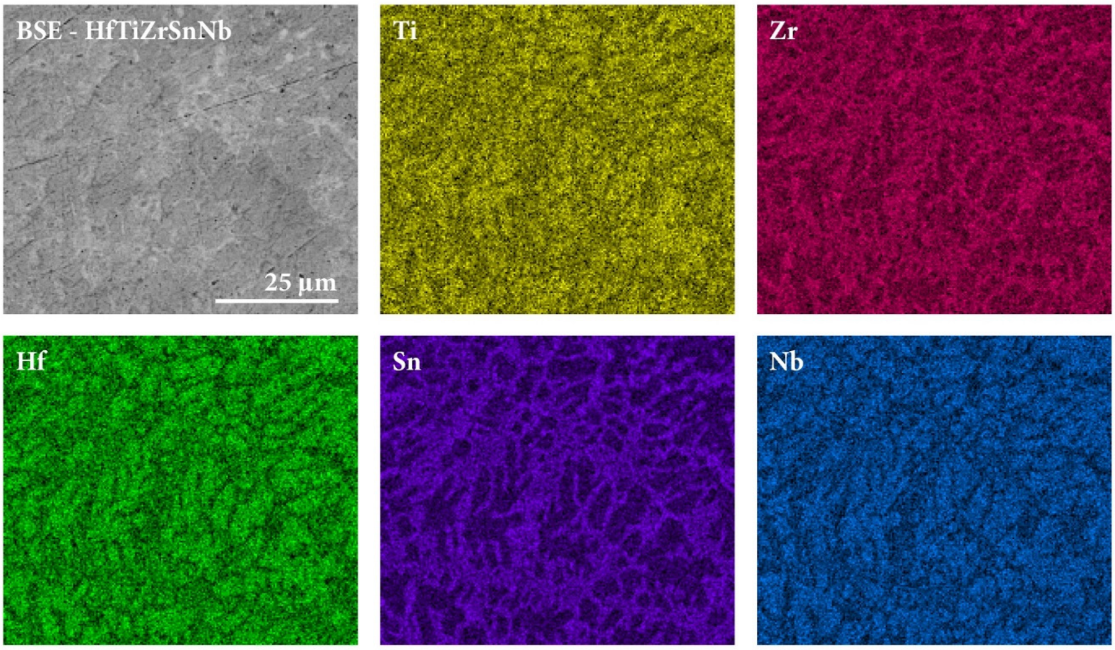

Supplement: Supplementary file 1 [file materials-14-03953-s001.zip › materials-1284587-supplementary.pdf]
